# Supplementary material for: Nearly one-third of lactating mothers are suffering from undernutrition in pastoral community, Afar region, Ethiopia: Community-based cross-sectional study
Source: PLoS One. 2021 Jul 9;16(7):e0254075. doi: 10.1371/journal.pone.0254075 (PMC8270417; doi:10.1371/journal.pone.0254075)
Supplement: S1 File — (DOCX) [file pone.0254075.s001.docx]

**Written Information Sheet and Consent Form**

**Introduction**

Hello, my name is ____________________. I am working as data collector for the study being conducted in this community by Samara University, College of Health Sciences. I kindly request you to lend me your attention to explain you about the study and being selected as the study participant.

**Study Title**

Nutritional status of lactating women and associated factors in pastoralist community, Aba’la district, Afar, Ethiopia: Community based cross sectional study: 2020.

**Purpose**

This study is intended to assess nutritional status of lactating women and associated factors in Aba’la district, region. Therefore, the information obtained from this study may be used by MOH, organizations supporting services in your community, researchers and local health planners for promotion of nutritional and other related intervention and prevention of factors contributing for maternal under nutrition in general.

**Procedure and duration**

First of all, you were selected by lottery method. I will be interviewing you using a questionnaire to provide me with pertinent data that is helpful for the study. There are questions to answer where I will fill the questionnaire by interviewing you and I will measure your height, weight and MUAC. The interview will take about 30 minutes, so I kindly request you to spare me this time for the interview and measurement.

**Risks**

The risks of being participating in this study are very minimal, but only taking few minutes from your time. Other than this the interview and measurement will not cause any physical harm on you and the community.

**Benefit**

There would not be any direct payment for participating in this study. But the findings from this research may reveal important information for the local health planners.

**Confidentiality**

The information that you provide us will be confidential. There will be no information that will identify you and your organization. The findings of the study will be general for the study population and will not reflect anything particular of individual persons or housing. The questioner will be coded to exclude showing names; no references will be made in oral or written reports that could link participants to the research.

**Rights**

Participation in this study is fully voluntary. You have the right to declare for your organization to participate in this study. You may refuse to answer any question or choose to stop the interview at any time. However, we hope you will answer the questions, which will benefit the services you provide and the nation.

**Contact address**

If there are any questions or enquires any time about the study, please contact and speak to principal investigator. Name: Getahun Fentaw Mulaw, Phone number: **+2519 10143645** and Email: [***gechfentaw1014@gmail.com***](mailto:gechfentaw1014@gmail.com).

**Statement of Consent**

I have read (was read to me) the participant information sheet. I have clearly understood the purpose of the research, the procedure, risks and benefits, issues of confidentiality, rights of participating and contact address for any queries. I have given the opportunity to ask questions for things that may have been unclear. I was informed that I have the right to withdraw from the study at any time or not to answer any question that I do not want. Therefore, I declare my voluntary consent to participate in this study with my initials (signature) as indicated below.

Participant's signature _________________date______/______/2020

Interviewer's name and signature _________________date _____/______/2020

May I begin the interview? **Yes** **No**

## **Study Tool / an Interview Questionnaire (English Version)**

| No | | **Part I:- Socio-demographic and socioeconomic characteristics** | | | | | | | | |
| --- | --- | --- | --- | --- | --- | --- | --- | --- | --- | --- |
| 001 | | What is the age of the index child | | | | | | __________(in complete months) |  | |
| 002 | | What is the sex of the index child? | | | | | | 1. Male 2. Female |  | |
| 003 | | What is your age (The mother)? | | | | | | ______ (In complete years) |  | |
| 004 | | How many are family members living together (family size)? | | | | | | __________ (in number) |  | |
| 005 | | How many numbers of under-five children are living in the household (including non-biological offspring)? | | | | | | __________ (in number) |  | |
| 006 | | Residence? | | | | | | 1. Rural 2. Urban |  | |
| 007 | | What is your ethnicity? | | | | | | 1. Afar 2. Tigray 3. Amhara 4. Other (specify)_______ |  | |
| 008 | | What is your religion? | | | | | | 1. Muslim 2. Orthodox 3. Protestant 4. Others(specify)_______ |  | |
| 009 | | What is your highest educational status (maternal)? | | | | | | 1. Unable to write & read 2. Able to read and write (informal education) 3. Grade 1-4 4. Grade 5-8 5. Grade 9-10 6. Grade 11-12 7. Certificate and diploma 8. Degree and above |  | |
| 010 | | What is your current occupation (maternal)? | | | | | | 1. Pastoralist 2. Student 3. Housewife 4. Government employee 5. Non-government employee 6. Merchant 7. Daily laborer 8. Other(Specify)_____ |  | |
| 011 | | What is your current marital status (maternal)? | | | | | | 1. Single 2. Married 3. Divorced 4. widowed 5. Other (specify)_____ |  | |
| 012 | | What is your husband’s highest educational status? | | | | | | 1. Unable to write & read 2. Able to read and write (informal education) 3. Grade 1-4 4. Grade 5-8 5. Grade 9-10 6. Grade 11-12 7. Certificate and diploma 8. Degree and above |  | |
| 013 | | What is your husband’s current occupation? | | | | | | 1. Pastoralist 2. Student 3. Government employee 4. Non-government employee 5. Merchant 6. Daily laborer 7. Other(Specify)______ |  | |
| 014 | | Who is the decision maker of the household? | | | | | | 1. Husband 2. Wife 3. Jointly 4. Other (specify)_____ |  | |
| 015 | | How much time it takes to reach the nearby health institution from household? | | | | | | __________(in minute ) |  | |
| 016 | | Do you have Functional TV/Radio? | | | | | | 1. Yes 2. No |  | |
| 017 | | Do you have livestock? | | | | | | 1. Yes 2. No |  | |
| 018 | | Did you have farm land? | | | | | |  |  | |
| **Part II: Maternal health care and obstetric history related factors** | | | | | | | | | | |
| 100 | | How many pregnancies have you had in your life (including the index child)? | | | | ________(in number) | | |  | |
| 101 | | How many of them are alive? | | | | ________(in number) | | |  | |
| 102 | | What was your age at marriage? | | | | _________(in complete years) | | |  | |
| 103 | | What was your age at first pregnancy? | | | | _________(in complete years) | | |  | |
| 104 | | Did you have ANC follow up for this child? | | | | 1. Yes 2. No | | |  | |
| 105 | | If yes how many times do you have ANC follow-up? | | | | 1. 1 times 2. 2 times 3. 3 times 4. >= 4times | | |  | |
| 106 | | How long is your preceding birth interval (in month) | | | | 1. First birth 2. ______(in complete month) | | |  | |
| 107 | | Where did you give birth (the index child)? | | | | 1. At health institution 2. At home 3. Others (specify)_______ | | |  | |
| 108 | | What was the way of delivery for the index child? | | | | 1. Spontaneous vaginal delivery 2. Caesarean section 3. Other (specify)______ | | |  | |
| 109 | | Was there any kind of pre delivery and delivery complications for the index child? | | | | 1. Yes 2. No | | |  | |
| 110 | | If yes what type of problem you faced? | | | | ________________ | | |  | |
| 111 | | Did you encounter recent history of recurrent infection (in the past two months)? | | | | 1. Yes 2. No | | |  | |
| 112 | | If yes how many times you get diseased in the last one year? | | | | ________(in number) | | |  | |
| 113 | | Do you believe that you have work load (maternal Judgment) | | | | 1. Yes 2. No | | |  | |
| **Part III: Maternal feeding practice** | | | | | | | | | | |
| 200 | | Did you receive nutrition related health education? | | | | 1. Yes 2. No | | |  | |
| 201 | | Did you take additional/ extra meal while you were pregnant? | | | | 1. Yes 2. No | | |  | |
| 202 | | If yes how many times per day? | | | | ________(in number) | | |  | |
| 203 | | Do you take additional/ extra meal during lactation? | | | | 1. Yes 2. No | | |  | |
| 204 | | If yes how many times per day? | | | | ___________(in number) | | |  | |
| 205 | | Is there any food taboo for the mother during pregnancy? | | | | 1. Yes 2. No | | |  | |
| 206 | | If yes list them | | | | ______________________ | | |  | |
| 207 | | Is there any food taboo for the mother during lactation? | | | | 1. Yes 2. No | | |  | |
| 208 | | If yes list them | | | | _______________ | | |  | |
| 209 | | What is the reason for not eating such foods during lactation? | | | | ______________________________________________ | | |  | |
| 210 | | **List all food and drinks the you(mother) taken during last 24 hour** | | | | | | | | |
|  |  | Breakfast |  | | | | | | | |
|  |  | Snuck |  | | | | | | | |
|  |  | Lunch |  | | | | | | | |
|  |  | Snuck |  | | | | | | | |
|  |  | Dinner |  | | | | | | | |
|  |  | Snuck |  | | | | | | | |
|  | | **Probing -Circle for the food item the mother consumes in the last 24 hour** | | | | | | | | |
|  | | **Food groups** | | **Specific food items** | | | | | | |
|  | | 1. Starchy staples | | Bread, Enjera, or any food made of maize, sorghum, millet, wheat, barley, teff) | | | | | | |
|  |  | 1. DGLVs | | Dark green leafy vegetables, including wild forms + locally available vitamin A rich leaves such as amaranth, cassava leaves, kale, spinach | | | | | | |
|  |  | 1. Other vitamin A rich fruits and vegetables | | Mango, Papaya. Banana , Peach, Carrot, Sweet potato, Lettuce, Pumpkin | | | | | | |
|  |  | 1. Other fruits and vegetables | | Tomato, onion, eggplant,other locally available  vegetables | | | | | | |
|  |  | 1. Organ meat | | liver, kidney, heart or other organ meats or blood-based foods | | | | | | |
|  |  | 1. Meat and fish | | Beef, pork, goat, ship, chicken | | | | | | |
|  |  | 1. Eggs | | Eggs | | | | | | |
|  |  | 1. Legumes, nuts & seeds | | Dried beans, dried peas, lentils, nuts, seeds or foods made from these (eg. hummus, peanut butter) | | | | | | |
|  |  | 1. Milk and milk products | | milk, cheese, yogurt or other milk products | | | | | | |
| **Part Iv : Hygiene and sanitation related characteristics** | | | | | | | | | | |
| 300 | | What is your source of water? | | | 1. Piped water 2. Inside the compound 3. Public stand 4. Spring water 5. Protected spring 6. Unprotected spring 7. Well , pond, river, lake, stream 8. protected 9. unprotected 10. Others(specify)_______ | | | | |  |
| 301 | | Do you have a latrine? | | | 1. Yes 2. No | | | | |  |
| 302 | | What type of latrine do you have? | | | 1. Pit latrine 2. Ventilated improved pit latrine(VIPL) 3. Other (specify)_________ | | | | |  |
| 303 | | Is there a hand washing basin with water at the door step of the latrine? | | | 1. Yes 2. No | | | | |  |
| 304 | | Is the latrine currently used by family members? | | | 1. Yes 2. No | | | | |  |
| 305 | | When did you wash your hand?(caregiver/ mother) | | | 1. Before food preparation 2. Before feeding herself 3. Before feeding the child 4. After toilet 5. After child cleaning 6. Using soap for hand washing | | | | |  |
| 306 | | Do you use soap or any disinfectant during hand washing | | | 1. Yes 2. No | | | | |  |
| 307 | | Where do you dispose solid waste? | | | 1. Open field inside the compound 2. Open field outside the compound 3. Private Pit 4. Municipality/communal pit 5. Other(specify)____________ | | | | |  |
| **Part V: Maternal anthropometric measurements** | | | | | | | | | | |
| 400 | Maternal height | | | | | | ___________(in meter) | | | |
| 401 | Maternal weight | | | | | | ___________(in kg) | | | |
| 402 | Mothers MUAC | | | | | | ___________(in cm) | | | |

**-----------------------Thank you -------------------**

**የመረጃ መሰብሰቢያ ቅጽ (በአማርኛ)**

**መግቢያ**

ጤና ይስጥልኝ፣ ስሜ____________ይባላል፡፡ እኔ በሰመራ ዩኒቨርሲቲ ጤና ሳይንስ ኮሌጅ በሚሰራዉ ጥናት እንደ መረጃ ሰብሳቢ ሆኘ እየሰራሁ ነው፡፡ ስለዚህ ስለጥናቱ እና እርሰዎ የተመረጡበትን ምክንያት ስገልፅለዎት በጥሞና እንዲከታተሉኝ በታላቅ አክብሮት እጠይቀወታለሁ፡፡

**የጥናቱ ርዕስ**፡- በአፋር ክልል በአብአላ ወረዳ የሚገኙ በወለዱ እናቶች ላይ ያለዉን የምግብ እጥረት እና የአመጋገብ ሁኔታ እነዲሁም በምን ምክንያት ሊከሰት እንደሚችል ለማወቅ የሚደረግ ጥናት

**የጥናቱ ዓላማ**፡- የዚህ ጥናት ዓላማ በወለዱ እናቶች ላይ ያለዉን የምግብ እጥረት እና የአመጋገብ ሁኔታ ለማወቅ እና በምን ምክንያት ሊከሰት እንደሚችል ለማወቅ የሚረዳ ሲሆን በዚህ ጥናት የሚገኘው ውጤትም ለጤና ጥበቃ ሚኒስተር ፣ ለድጋፍ ሰጪ ድርጅቶች፣ ለጥናት ባለሙያዎች እና ለአካባቢው የጤና እቅድ አዉጭ አካላት መረጃው ከደረሳቸው በኋላ የጥናቱን ውጤት አስመልክተው ለበለጠ ዕቅድ ለማቀድ ይጠቅማቸዋል፡፡

**የጥናቱ ሂደት እና ጊዜ**፡- በመጀመርያ እርሶ የተመረጡት በዕጣ ነው፡፡ አሁን የምጠይቀዎት ይሄንን ልጅ ካረገዝሽ ጀምሮ ያሉትን ሁኔታወች በተመለከተ ጥናቱን የሚረዳ ትክክለኛውን መረጃ እንዲሰጡኝ ነዉ፡፡ ባማካኝ ዉይይታችን ከ 20-30 ደቂቃ ሊጨርስ ይችላል እናም መጠይቁን የምሞላው ጥያቄዎቹን እየጠየቅኩ እና የእርስዎን ክብደት፣ ቁመትና የእጀዎን ዙሪያ መጠን በመለካት ነው፡፡ ስለሆነም ይህንን መረጃ ለመስጠት ጊዜዎትን በመስጠት እንዲተባበሩን በአክብሮት እጠይቃለሁ፡፡

**ጉዳት፡-** በዚህ ጥናት በመሳተፍዎ ያለው ጉዳት በጣም አነስተኛ ነው፡፡ ከእረፍት ግዜዎ ላይ ጥቂት ደቂቃ ሊወስድ ይችላል፡፡ ከዚህ በተረፈ ጥናቱ በእርሶም ሆነ በህብረተሰቡ ላይ ምንም ጉዳት አያደርስም፡፡

**ጥቅም፡-** በዚህ ጥናት በመሳተፍዎ ቀጥተኛ ክፍያ ላያገኙ ይችላሉ ግን የጥናቱ ዉጤት ለአካባቢው የጤና እቅድ አዉጭ አካላት መረጃው ጠቃሚ መረጃ ሊሰጥ ይችላል፡፡

**ሚስጥር አጠባበቅ፡-** የሚሰጡን መረጃ ሁሉ ሚስጥርነቱ የተጠበቀ ነው፡፡ ለዚሁም እርሶነትዎን እና ቤተሰብዎን የሚገልጽ ምንም ነገር የለም፡፡ የጥናቱ ውጤት ለግለሰብ ወይም ደግሞ ለቤት ብቻ ሳይሆን ለአጠቃላይ ህብረተሰብ የሚውል ይሆናል፡፡ ጥያቄው በሚስጥር ፅሁፍ ስለሆነ ምንም የእርሶን መልስ ከእረሶ ጋር በቃል ወይም በጽሁፍ የሚያያዝ ነገር አይኖርም፡፡

**የተሳታፊው መብት፡-** በዚህ ጥናት ለመሳተፍ ሙሉ በሙሉ በፈቃደኝነት ነው፡፡ በዚህ ጥናት መሳተፍዎን ለቤተሰብዎ የመግለጽ መብት አለዎት፡፡ ማንኛዉም ጥያቄ አለመመለስ ይችላሉ ወይም ለመሳተፍ ካልፈለጉ ደግሞ በማንኛውም ጊዜ ራስዎን ከጥናቱ ማግለል (ማቋረጥ) ይችላሉ፡፡ ሆኖም ግን ለእርሶ እና ለህዝብ ጥቅም ሲሉ ጥናቱ ላይ እንደሚሳተፉ ተስፋ እናደርጋለን፡፡

**አድራሻ፡-** ጥናቱን በተመለከተ ማንኛውም ጥያቄ ካለዎት በየትኛዉም ሰዓት የጥናቱን መሪ በሚከተለው አድራሻ ማግኘትና ማነጋገር ይችላሉ፡፡ ስም፡ **ጌታሁን ፈንታዉ**፣ ስ.ቁ: **+251910143645** እና Email: [gechfentaw1014@gmail.com](mailto:gechfentaw1014@gmail.com)

**የስምምነት ማረጋገጫ ቅፅ**

የተሳታፊው መረጃ ቅፅ አንብቤዋለሁ (ተነቦልኛል)፡፡ የጥናቱ ዓላማ ፣ ያለውን ጉዳት እና ጥቅም ፣ ምስጢር አጠባበቅ፣ የመሳተፍ እና ያለመሳተፍ መብት እንዲሁም ችግር ካለ (ቢፈጠር) ከማን ጋር መገናኘት እንዳለብኝ ሁሉ ተገልጾልኝ ጥያቄ ካለኝ ደግሞ እንድጠይቅ እድል ተሰጥቶኝ በመሀል ደግሞ ጥናቱን ለማቆም ከፈለኩኝ በማንኛውም ጊዜ ከጥናቱ/ከተሳታፊነት/ መውጣት እንደምችል በመጨረሻም መመለስ የማልፈልገውን ጥያቄ አለመመለስ መብት እንዳለኝ ከተረዳሁኝ በኋላ በሙሉ ፈቃደኝነት በዚህ ጥናት ለመሳተፍ የወሰንኩኝ መሆኔን ከዚህ በታች በተቀመጠው ፊርማዬ አረጋግጣለሁ፡፡

የተሳታፊው ፊርማ__________________ ቀን _______/______/2011ዓ.ም

የመረጃ ሰብሳቢ ስም እና ፊርማ_____________________ ________ቀን _____/_____/2011ዓ.ም

ጥያቄዎችን መጠየቅ ልጀምር? **አዎን** **አይ**

**የመጠይቁ መለያ ቁጥር: _____________ ቀበሌ___________**

| No | **ክፍል 1፡ ማህበራዊና ኢኮኖሚያዊ ሁኔታዎችን በተመለከተ** | | | | | | | | | | |
| --- | --- | --- | --- | --- | --- | --- | --- | --- | --- | --- | --- |
| 001 | የመጨረሻ ልጅሽ እድሜ ስንት ነዉ? | | | | | __________(በወር) | | | |  | |
| 002 | የህፃኑ/ኗ ፆታ | | | | | 1. ወንድ 2. ሴት | | | |  | |
| 003 | እድሜሽ ስንት ነዉ? | | | | | __________(በኣመት) | | | |  | |
| 004 | አንድ ላይ የምትኖሩ የቤተሰብ አባላት ብዛት ስንት ናቹህ ? | | | | | __________ (በቁጥር) | | | |  | |
| 005 | ቤታቹህ ዉስጥ ከ 5 አመት በታች ያሉ ህፃናት ብዛት ስንት ናቸዉ? | | | | | __________ (በቁጥር) | | | |  | |
| 006 | የሚኖሩበት አካባቢ ከተማ ነዉ ወይንስ ገጠር? | | | | | 1. ገጠር 2. ከተማ | | | |  | |
| 007 | ብሄርሽ ምንድን ነዉ? | | | | | 1. አፋር 2. ትግሬ 3. አማራ 4. ሌላ (ካለ ይገለፅ) ________ | | | |  | |
| 008 | የትኛዉን ሀይማኖት ነዉ የምትከተይዉ? | | | | | 1. ሙስሊም 2. ኦርቶዶክስ 3. ፕሮቴስታንት 4. ሌላ (ካለ ይገለፅ) ________ | | | |  | |
| 009 | አሁን ያለሽ ከፍተኛ የትምህርት ደረጃ ምን ያክል ነዉ? | | | | | 1. ማንበብ መፃፍ የማትችል 2. ማንበብ መፃፍ የምችል(ኢ-መደበኛ) 3. ከ1-4 ክፍል የጨረሰች 4. ከ 5-8 ክፍል የጨረሰች 5. ከ 9-10 ክፍል የጨረሰች 6. ከ 11-12 ክፍል የጨረሰ 7. ሰርቲፊኬት እና ዲፕሎማ 8. ዲግሪ እና ከዛ በላይ | | | |  | |
| 010 | አሁን የምሰሪዉ የስራ አይነት ምንድን ነዉ? | | | | | 1. አርብቶ አደር 2. ተማሪ 3. የቤት እመቤት 4. የመንግስት ሰራተኛ 5. መንግስታዊ ያልሆነ ሰራትኛ 6. ነጋዴ 7. የቀን ሰራተኛ 8. ሌላ (ይገለፅ)___________ | | | |  | |
| 011 | የጋብቻ ሁኔታሽ ምን ይመስላል? | | | | | 1. ያገባች 2. ያላገባች 3. የፈታች 4. ባሏ የሞተባት 5. ሌላ (ይገለፅ)___________ | | | |  | |
| 012 | የባለቤትሽ አሁን ያለበት ከፍተኛ የትምህርት ደረጃ ስንት ነዉ? | | | | | 1. ማንበብ መፃፍ የማይችል 2. ማንበብ መፃፍ የሚችል (ኢ-መደበኛ) 3. ከ 1-4 ክፍል የጨረስ 4. ከ 5-8 ክፍል የጨረሰ 5. ከ 9-10 ክፍል የጨረሰ 6. ከ 11-12 ክፍል የጨረሰ 7. ሰርቲፊኬት እና ዲፕሎማ 8. ዲግሪ እና ከዛ በላይ | | | |  | |
| 013 | የባለቤትሸ አሁን የሚሰራዉ የስራ አይነት ምንድን ነዉ? | | | | | 1. አርብቶ አደር 2. ተማሪ 3. የመንግስት ሰራተኛ 4. መንግስታዊ ያልሆነ ሰራትኛ 5. ነጋዴ 6. የቀን ሰራተኛ 7. ሌላ (ይገለፅ)___________ | | | |  | |
| 014 | የቤተሰብ ዉሳኔ ሰጭ አካል ማነዉ? | | | | | 1. ባል 2. ሚስት 3. ባልና ሚስት አንድ ላይ 4. ሌላ (ይገለፅ)___________ | | | |  | |
| 015 | ከቤታቹህ ወደ አቅራቢያቹህ የሚገኘዉ ጤና ተቋም ለመድረስ ምን ያክል ግዜ ይወስዳል? | | | | | _________(በደቂቃ) | | | |  | |
| 016 | አሁን እየተጠቀማቹህበት ያለ የሚሰራ ቴሌቪዥን ወይንም ሬዲዮ አላቹህ? | | | | | 1. አወ 2. የለንም | | | |  | |
| 017 | የቤት እነሰሳት (ግመል፣ ከብት፣ ፍየል፣ በግ፣ደሮ) አላቹህ? | | | | | 1. አወ 2. የለንም | | | |  | |
| 018 | የእርሻ መሬት አላቹህ? | | | | | 1. አወ 2. የለንም | | | |  | |
| **ክፍል 2፡ የእናትየዋ የጤና አጠባበቅን በተመልከተ** | | | | | | | | | | | |
| 100 | እስካሁን ስንት እረግዝናዎች ነበሩሽ? | | | | | | __________(በቁጥር) | | |  | |
| 101 | በሂዎት ያሉት ስንት ናቸዉ? | | | | | | _________(በቁጥር) | | |  | |
| 102 | መጀመርያ ስታገቢ እድሜሽ ስንት ነበር? | | | | | | _________(በዓመት) | | |  | |
| 103 | መጀመርያ ስታረግዥ እድሜሽ ስንት ነበር? | | | | | | __________(በዓመት) | | |  | |
| 104 | ይህን ህፃን አርግዘሽ እያለ የቅድመ ወሊድ ክትትል ታደርጊ ነበር? | | | | | | 1. አወ 2. አላደርግም | | |  | |
| 105 | ክትትል የምታደርጊ ከነበር ስንት ግዜ አድርገሽ ነበር? | | | | | | ___________(በቁጥር) | | |  | |
| 106 | በመጨረሻ ሁለቱ ልጆችሽ መካከል ያለዉ የእድሜ ልዩነት (ግዜ) ምን ያክል ነዉ? | | | | | | 1. የመጀመርያ ልጄ ነዉ 2. ____________(በወር) | | |  | |
| 107 | ይህንን ልጅሽን የት ነዉ የወለሽዉ? | | | | | | 1. በጤና ተቋም 2. ቤት 3. ሌላ (ይገለፅ)________ | | |  | |
| 108 | ልጁን እንዴት ነዉ የወለድሽዉ? | | | | | | 1. በምጥ 2. በቀዶ ጥገና 3. ሌላ (ይገለፅ)________ | | |  | |
| 109 | ይህንን ህፃን ስትወልጅ ወይንም ከዛ በፊት ያገጠመሽ ችግር ነበር? | | | | | | 1. አወ 2. አላጋጠመኝም | | |  | |
| 110 | አወ ከሆነ ምን አይነት ችግር ነበር ያጋጠመሽ? | | | | | | _______________ | | |  | |
| 111 | ባለፈዉ 1 አመት ዉስጥ ታመሽ ታዉቂያሽ? | | | | | | 1. አወ 2. አሞኝ አያቅም | | |  | |
| 112 | አወ ከሆን በያንስ ስንት ግዜ ታመሽ ታዉቂያለሽ? | | | | | | _________(በቁጥር) | | |  | |
| 113 | የስራ ጫና አለብኝ ብለሽ ታስቢያለሽ? | | | | | | 1. አወ 2. የለብኝም | | |  | |
| **ክፍል 3፡ ስለእናትየዋ የአመጋገብ ስርዓት በተመለከተ** | | | | | | | | | | | |
| 200 | ስለጤናማ አመጋገብ ትምህርት አግተሸ ታዉቂያለሽ? | | | | | | 1. አወ 2. አላዉቅም | | |  | |
| 201 | በእርግዝና ግዜ ከተለመደዉ የአመጋገብ ዘዴ በተለየ ተጨማሪ ምግብ ትወስጅ ነበር? | | | | | | 1. አወ 2. አልወስድም | | |  | |
| 202 | አወ ከሆነ ምን ያክል ግዜ ተጨማሪ ምግብ ትወስጃለሽ? | | | | | | ________(በቁጥር) | | |  | |
| 203 | በወሊድ (ልጅ በምታጠቢበት) ግዜ ከተለመደዉ የአመጋገብ ዘዴ በተለየ ተጨማሪ ምግብ ትወስጃለሽ? | | | | | | 1. አወ 2. አልወስድም | | |  | |
| 204 | አወ ከሆነ ምን ያክል ግዜ ተጨማሪ ምግብ ትወስጃለሽ? | | | | | | ________(በቁጥር) | | |  | |
| 205 | በባህላቹሀ ነብሰ-ጡር እናት በጭራሽ እንዳትበላ የሚከለከል የምግብ አይነት አለ (food taboo)? | | | | | | 1. አወ 2. የለም | | |  | |
| 206 | አወ ከሆነ ምን አይነት ምግብ ነዉ? | | | | | | ________________ | | |  | |
| 207 | በባህላቹሀ የወለደች እናት በጭራሽ እንዳትበላ የሚከለከል የምግብ አይነት አለ (food taboo)? | | | | | | 1. አወ 2. የለም | | |  | |
| 208 | አወ ከሆነ ምን አይነት ምግብ ነዉ የሚከለከለዉ? | | | | | | __________________ | | |  | |
| 209 | ምግቡ የሚከለከልበት ምክንያት ለምንድን ነዉ? | | | | | | __________________ | | |  | |
| 210 | በለፈዉ 24 ስዓት ዉስጥ የተጠቀምሻቸዉን የምግብና የመጠጥ አይነት በማስታወስ በቅደም ተከተል ይንገሩኝ (ትላንት የተለየ ቀን ከነበረ ከትላንት በፊት የነበረዉን ቀን ይንገሩኝ) | | | | | | | | | | |
|  | **ቁርስ** |  | | | | | | | | | |
|  | **ማራፈጃ** |  | | | | | | | | | |
|  | **ምሳ** |  | | | | | | | | | |
|  | **መክሰስ** |  | | | | | | | | | |
|  | **እራት** |  | | | | | | | | | |
|  | **ከራት ቡሃላ (ሙሉ ለሊቱን)** |  | | | | | | | | | |
|  | እናትየዋ ባለፈዉ 24 ስዓት ዉስጥ የተጠቀመቻቸዉን የምግብ አይነቶች እንድታስታዉስ የሚረዱ መጠይቆች (ከዚህ የተጠቀሱትን ዝርዝር የምግብ አይነቶች ለእናትዮዋ በማንበብ፡ **ወስዳ ከሆነ 1 ፣ ካልወሰደች 2** እያሉ እያንዳነዱ የምግብ ዝርዝር ፊት ይፃፉ) | | | | | | | | | | |
|  | **የምግብ አይነቶች** | | | **ዝርዝር ምግቦች** | | | | | | | 1. **አወ** 2. **አልወሰድኩም** |
|  | 1. ሀይል ሰጪ ምግቦች | | | ዳቦ፣ እንጀራ፣ ወይንም ሌላ የምግብ አይነቶች ከ ጤፍ፣ ገብስ፣ስንዴ፣ ማሽላ፣ በቀሎ፣ የተሰሩ | | | | | | |  |
|  | 1. አረንጓዴ የአትክልት አይነቶች | | | አረንጓዴ የሆኑ የአትክልት አይነቶች እና በ ቫይታሚን-A የበለፀጉ አትክልቶች ለምሳሌ ሰላጣ፣ጎመን | | | | | | |  |
|  | 1. በ ቫይታሚን -A የበለፀጉ ፍራፍሬዎችና አትክልቶች | | | ማንጎ፣ ፓፓየ፣ ሙዝ፣ አፕል፣ ካሮት፣ ስኩር ድንች፣ ጥቅል ጎመን፣ ዱባ | | | | | | |  |
|  | 1. ሌላ ፍራፍሬዎችና አትክልቶች | | | ቲማቲም፣ ሽንኩርት፣ እና በአካባቢዉ የሚገኑ እነዚህን የመሳሰሉ አትክልቶች | | | | | | |  |
|  | 1. ዋናዋና የሰዉነት ክፍል የስጋ አይነቶች (organ meat) | | | ጉበት፣ ኩላሊት፣ ልብ እና ሌላ የተለያዩ በደም የበለፀጉ የእንሰሳ አካል ምግቦች | | | | | | |  |
|  | 1. የስጋ አይነቶች | | | ስጋ (የከብት፣ የፍየል፣ የበግ፣ የደሮ፣ዓሳ) | | | | | | |  |
|  | 1. እንቁላል | | | እንቁላል | | | | | | |  |
|  | 1. የወጥ እና የመሳሰሉ ግዓቶች | | | ባቄላ፣አተር፣ምስር፣ ለዉዝ፣ እና ከነዚህ የተስሩ የምግብ፣ የወጥና የዘይት አይነቶች | | | | | | |  |
|  | 1. ወተት፣ እና ወተት ተዋፆኦች | | | ወተት፣ እና ወተት ተዋፆኦች | | | | | | |  |
| **ክፍል-4: -የግል ንፅህና እና የአከባቢ ፅዳት ጋር ተያያዥነት ያላቸዉ ጉዳዮች** | | | | | | | | | | | |
| 300 | ለመጠጥ የሚሆን ዉሃ የምታገኙት ከየት ነዉ? | | 1. ከቧንቧ    1. ግቢ ዉስጥ    2. የጋራ/የህዝብ 2. ከምንጭ    1. የተጠበቀ    2. ያልተጠበቀ 3. ጉድጓድ፣ ኩሬ፣ ወንዝ፣ ሃይቅ    1. የተጠበቀ    2. ያልተጠበቀ) 4. ሌላ (ይጥቀሱ)__________ | | | | | |  | | |
| 301 | ሽንት ቤት አላችሁ? | | | | 1. አዎ 2. የለንም | | | |  | | |
| 302 | ሽንት ቤታችሁ ምን ዓይነት ነዉ? | | | | 1. ባህላዊ የጉድጓድ 2. ዘመናዊ 3. ሌላ (ይጥቀሱ)__________ | | | |  | | |
| 303 | የሽንት ቤቱ በር አከባቢ የእጅ መታጠቢያ ዉሃ አለ? | | | | 1. አዎ 2. የለንም | | | |  | | |
| 304 | ሽንት ቤቱ አሁን ላይ ትጠቀሙታላችሁ? | | | | 1. አዎ 2. አንጠቀመዉም | | | |  | | |
| 305 | እጅሽን የምትታጠቢዉ መቸ ነዉ (እናት)? | | 1. ምግብ ከማዘጋጀት በፊት 2. ምግብ ከመመገቤ በፊት 3. ህጻኑን ምግብ ከመመገቤ በፊት 4. ሽንት ቤት ከተጠቀምኩ በኃላ 5. ልጄን ከአጸዳዳሁ በኃላ 6. ልል (ካላ ይግለጹ)______________ | | | | | |  | | |
| 306 | እጅዎን ሰትጣጠቡ ሳሙና ይጠቀማሉ? | | 1. አዎ 2. አልጠቀምም | | | | | |  | | |
| 307 | ደረቅ ቆሻሻ የምትጥሉት የት ነዉ? | | 1. ሜዳ ላይ (ግቢ ዉስጥ) 2. ሜዳ ላይ (ከግቢ ዉጪ) 3. ግቢ ዉስጥ ያለ ጉድጓድ ዉስጥ 4. ማዘጋጃ ቤት/ቀበሌ ያዘጋጀዉ ጉድጓድ ውስጥ 5. ሌላ (ይጥቀሱ)__________ | | | | | |  | | |
| **ክፍል 5፡ የእናትየዋ የክብደት፣ ቁመት እና ሌሎች ልኪቶች** | | | | | | | | | | | |
| 400 | የእናትዮዋ ቁመት | | | | | | | ___________(በ ሜትር) | | | |
| 401 | የእናትዮዋ ክብደት | | | | | | | ___________(በ ኪ.ግ) | | | |
| 402 | የእናትዮዋ መካከለኛ የእጅ ዙሪያ (MUAC) | | | | | | | ____________ በ ሴ.ሜ | | | |

…………………**...እናመሰግናለን**!!!..................
