# Supplementary material for: Nearly one-third of lactating mothers are suffering from undernutrition in pastoral community, Afar region, Ethiopia: Community-based cross-sectional study
Source: PLoS One. 2021 Jul 9;16(7):e0254075. doi: 10.1371/journal.pone.0254075 (PMC8270417; doi:10.1371/journal.pone.0254075)
Supplement: Attachment — Submitted filename: PONE-D-20-31073_R1-CG Nutrition lactating mothers.pdf [file pone.0254075.s004.pdf]

PLOS ONE

Nearly One-Third of Lactating Mothers are Suffering from Undernutrition in Pastoral Community, Afar Region, Ethiopia: Community-Based Cross-Sectional Study

--Manuscript Draft--

**Manuscript Number:**	PONE-D-20-31073R1
**Article Type:**	Research Article
**Full Title:**	Nearly One-Third of Lactating Mothers are Suffering from Undernutrition in Pastoral Community, Afar Region, Ethiopia: Community-Based Cross-Sectional Study
**Short Title:**	Nearly One-Third of Lactating Mothers are Suffering from Undernutrition in Pastoral Community, Ethiopia.
**Corresponding Author:**	Getahun Fentaw Mulaw, MPH<br>Woldia University<br>Woldia, Amhara ETHIOPIA
**Keywords:**	Undernutrition, lactating mothers, Abala district, Pastoralist, Afar, Ethiopia
**Abstract:**	Background: - Undernutrition is responsible for a significant proportion of maternal and child morbidity and mortality. Lactating women are nutritionally vulnerable groups because this period places a high nutritional demand on the mother and leads to nutritional stress. Poor nutrition during lactation has a significant negative consequence to mothers and children's survival, growth, and development. Therefore this study aimed to assess the nutritional status of lactating mothers and associated factors in pastoral community, Afar region, Ethiopia.<br>Methods: - A community-based cross-sectional study was conducted from January 5/2020 to February 10/2020, in the Abala district. The data were collected from a sample of 366 lactating mothers whose children aged less than 24 months. Data was collected through face-to-face interviews and anthropometric measurements. Study participants were recruited using a systematic sampling technique. Anthropometric measurements (both body mass index and mid-upper arm circumference) were taken from each mother using calibrated equipment and standardized techniques. Data were entered into Epi-data version 4.2 and exported to SPSS version 22 for analysis. Predictor variables with a P-value < 0.25 at bivariable analysis were candidates for the final model. Statistical significance was declared at P-value of < 0.05 in the multivariable logistic regression.<br>Result: - This study showed that 120(32.8%) and 122(33.3%) surveyed mothers were undernourished using a cut-off body mass index <18.5 kg/m2 and mid-upper arm circumference <23 Centimeter, respectively. Lactating mothers who didn't meet the minimum dietary diversity score were more than five (Adjusted odds ratio (AOR) = 5.103; 95% confidence interval (CI): 2.128, 12.238) times more likely to be undernourished than those who met the minimum dietary diversity score. Mothers with short birth intervals were also more than four (AOR=4.800; 95% CI: 2.408, 9.567) time more likely to be undernourished.<br>Conclusion: - Nearly one-third of lactating mothers were undernourished. Undernutrition among lactating mothers was significantly associated with maternal dietary diversity score and birth interval. Health education on proper and adequate maternal dietary feeding practices, and proper family planning utilization during lactation should be emphasized.
**Order of Authors:**	Getahun Fentaw Mulaw, MPH
	kusse Urmale Marie
	Etsay Woldu Anbesu
**Opposed Reviewers:**	
**Response to Reviewers:**	Comments from the Editor: It was amazing to get such like professional comments from the editor; I accepted the comments and tried to amend them.<br><br>Comments from reviewer one: Thank you for your detail comments and suggestions. You have touched each part of the manuscript in detail, and accordingly I modified the whole document.

Powered by Editorial Manager® and ProduXion Manager® from Aries Systems Corporation

	Comments from reviewer two: Thanks for constructive general comments, which indicate me to deal more on the manuscript
**Additional Information:**	
**Question**	**Response**
**Financial Disclosure**<br><br>Enter a financial disclosure statement that describes the sources of funding for the work included in this submission. Review the submission guidelines for detailed requirements. View published research articles from *PLOS ONE* for specific examples.<br><br>This statement is required for submission and **will appear in the published article** if the submission is accepted. Please make sure it is accurate.<br><br>**Unfunded studies**<br>Enter: *The author(s) received no specific funding for this work.*<br><br>**Funded studies**<br>Enter a statement with the following details:<br>• Initials of the authors who received each award<br>• Grant numbers awarded to each author<br>• The full name of each funder<br>• URL of each funder website<br>• Did the sponsors or funders play any role in the study design, data collection and analysis, decision to publish, or preparation of the manuscript?<br>• **NO** - Include this sentence at the end of your statement: *The funders had no role in study design, data collection and analysis, decision to publish, or preparation of the manuscript.*<br>• **YES** - Specify the role(s) played.<br><br>* typeset	he funders had no role in study design, data collection and analysis, decision to publish, or preparation of the manuscript.
**Competing Interests**<br><br>Use the instructions below to enter a competing interest statement for this submission. On behalf of all authors, disclose any competing interests that could be perceived to bias this work—acknowledging all financial support	The authors have declared that no competing interests exist.

and any other relevant financial or non-financial competing interests.<br><br>This statement **will appear in the published article** if the submission is accepted. Please make sure it is accurate. View published research articles from [*PLOS ONE*] for specific examples.<br><br>**NO authors have competing interests**<br><br>Enter: *The authors have declared that no competing interests exist.*<br><br>**Authors with competing interests**<br><br>Enter competing interest details beginning with this statement:<br><br>*I have read the journal's policy and the authors of this manuscript have the following competing interests: [insert competing interests here]*<br><br>* typeset	
**Ethics Statement**<br><br>Enter an ethics statement for this submission. This statement is required if the study involved:<br><br>• Human participants<br>• Human specimens or tissue<br>• Vertebrate animals or cephalopods<br>• Vertebrate embryos or tissues<br>• Field research<br><br>Write "N/A" if the submission does not require an ethics statement.<br><br>General guidance is provided below. Consult the submission guidelines for detailed instructions. **Make sure that all information entered here is included in the Methods section of the manuscript.**	The study protocol was approved by the Institutional Ethical Review Committee of Samara University; College of Health and Medical Science (ERC0053/2019). Written informed consent was obtained from lactating mothers, and confidentiality was also assured

**Format for specific study types**<br><br>**Human Subject Research (involving human participants and/or tissue)**<br>• Give the name of the institutional review board or ethics committee that approved the study<br>• Include the approval number and/or a statement indicating approval of this research<br>• Indicate the form of consent obtained (written/oral) or the reason that consent was not obtained (e.g. the data were analyzed anonymously)<br><br>**Animal Research (involving vertebrate animals, embryos or tissues)**<br>• Provide the name of the Institutional Animal Care and Use Committee (IACUC) or other relevant ethics board that reviewed the study protocol, and indicate whether they approved this research or granted a formal waiver of ethical approval<br>• Include an approval number if one was obtained<br>• If the study involved *non-human primates*, add *additional details* about animal welfare and steps taken to ameliorate suffering<br>• If anesthesia, euthanasia, or any kind of animal sacrifice is part of the study, include briefly which substances and/or methods were applied<br><br>**Field Research**<br><br>Include the following details if this study involves the collection of plant, animal, or other materials from a natural setting:<br>• Field permit number<br>• Name of the institution or relevant body that granted permission	
**Data Availability**<br><br>Authors are required to make all data underlying the findings described fully available, without restriction, and from the time of publication. PLOS allows rare exceptions to address legal and ethical concerns. See the PLOS Data Policy and FAQ for detailed information.	Yes - all data are fully available without restriction

A Data Availability Statement describing where the data can be found is required at submission. Your answers to this question constitute the Data Availability Statement and **will be published in the article**, if accepted.<br><br>**Important:** Stating 'data available on request from the author' is not sufficient. If your data are only available upon request, select 'No' for the first question and explain your exceptional situation in the text box.<br><br>Do the authors confirm that all data underlying the findings described in their manuscript are fully available without restriction?	
**Describe where the data may be found in full sentences. If you are copying our sample text, replace any instances of XXX with the appropriate details.**<br><br>• If the data are **held or will be held in a public repository**, include URLs, accession numbers or DOIs. If this information will only be available after acceptance, indicate this by ticking the box below. For example: *All XXX files are available from the XXX database (accession number(s) XXX, XXX.).*<br>• If the data are all contained **within the manuscript and/or Supporting Information files**, enter the following: *All relevant data are within the manuscript and its Supporting Information files.*<br>• If neither of these applies but you are able to provide **details of access elsewhere**, with or without limitations, please do so. For example:<br><br>*Data cannot be shared publicly because of [XXX]. Data are available from the XXX Institutional Data Access / Ethics Committee (contact via XXX) for researchers who meet the criteria for access to confidential data.*<br><br>*The data underlying the results presented in the study are available from (include the name of the third party*	All relevant data are within the manuscript and its Supporting Information files.

and contact information or URL).<br>• This text is appropriate if the data are owned by a third party and authors do not have permission to share the data.<br><br>* typeset	
Additional data availability information:	

From: Getahun Fentaw Mulaw

- Email: gechfentaw1014@gmail.com /getahun.f@wldu.edu.et
- ORCID: https://orcid.org/0000-0002-4173-3759
- Cell phone: +251910143645
- POBOX: 400
- Fax: +251335400643
- Wodia University Web Address: www: http://www.woldu.edu.et
- **Date: April 24/2021**

To: PLOS ONE

Subject: Submission of revised manuscript on nutritional status of lactating mothers (Manuscript ID: PONE-D-20-31073)

Dear Editorial team,

We had submitted a manuscript entitled " **Nearly One-Third of Lactating Mothers are Suffering from Under-nutrition in Pastoral Community, Afar Region, Ethiopia: Community-Based Cross-Sectional Study**" to your reputable journal.

First of all we would like to thank a lot for your invaluable constructive comments and suggestions for the improvement of this manuscript. Based on your interesting suggestion we, the authors, critically amend the whole manuscript document (as highlighted by red colors in the revised manuscript document).

Therefore we expect you to consider this research article for publication which will help for policy and program implementers, and also for future evidences for many scholars and other stakeholders.

Funding Statement: - The funder (Samara University) has no role in designing the study, data collection, data analysis, decision to publish, and preparation of the manuscript.

Kind regards

Getahun Fentaw Mulaw

(Lecturer and Researcher)

Manuscript Click here to access/download;Manuscript;Manuscript.docx

Nearly One-Third of Lactating Mothers are Suffering from Undernutrition in Pastoral Community, Afar Region, Ethiopia: Community-Based Cross-Sectional Study

Getahun Fentaw Mulaw*[1], Kusse Urmale Mare[2], Etsay woldu Anbesu[3]

[1]School of Public Health, College of Health Sciences, Woldia University, Woldia, Amhara region, Ethiopia

[2]Nursing Department, College of Health Sciences, Samara University, Samara, Afar region, Ethiopia

[3]Public Health Department, College of Health Sciences, Samara University, Samara, Afar region, Ethiopia

***Corresponding author:** *GFM*; Email:gechfentaw1014@gmail.com; ORCID: https://orcid.org/0000-0002-4173-3759)

Abstract

Background: - Undernutrition is responsible for a significant proportion of maternal and child morbidity and mortality. Lactating women are nutritionally vulnerable groups because this period places a high nutritional demand on the mother and leads to nutritional stress. Poor nutrition during lactation has a significant negative consequence to mothers and children's survival, growth, and development. Therefore this study aimed to assess the nutritional status of lactating mothers and associated factors in pastoral community, Afar region, Ethiopia.

Methods: - A community-based cross-sectional study was conducted from January 5/2020 to February 10/2020, in the Abala district. The data were collected from a sample of 366 lactating mothers whose children aged less than 24 months. Data was collected through face-to-face interviews and anthropometric measurements. Study participants were recruited using a systematic sampling technique. Anthropometric measurements (both body mass index and mid-upper arm circumference) were taken from each mother using calibrated equipment and standardized techniques. Data were entered into Epi-data version 4.2 and exported to SPSS version 22 for analysis. Predictor variables with a P-value < 0.25 at bivariable analysis were candidates for the final model. Statistical significance was declared at P-value of < 0.05 in the multivariable logistic regression.

Result: - This study showed that 120(32.8%) and 122(33.3%) surveyed mothers were undernourished using a cut-off body mass index <18.5 kg/m^2 and mid-upper arm circumference <23 Centimeter, respectively. Lactating mothers who didn't meet the minimum dietary diversity score were more than five (Adjusted odds ratio (AOR) = 5.103; 95% confidence interval (CI): 2.128, 12.238) times more likely to be undernourished than those who met the minimum dietary diversity score. Mothers with short birth intervals were also more than four (AOR=4.800; 95% CI: 2.408, 9.567) time more likely to be undernourished.

Conclusion: - Nearly one-third of lactating mothers were undernourished. Undernutrition among lactating mothers was significantly associated with maternal dietary diversity score and birth interval. Health education on proper and adequate maternal dietary feeding practices, and proper family planning utilization during lactation should be emphasized.

Keywords: - Undernutrition, lactating mothers, Abala district, Pastoralist, Afar, Ethiopia

Background

Malnutrition is one of the most devastating problems worldwide and can be manifested as both undernutrition and overnutrition. Undernutrition can be divided into protein-energy malnutrition and micronutrient deficiencies [1]. Undernutrition is the outcome of insufficient food intake, inadequate care, and infectious diseases [2, 3]. Malnutrition among reproductive-age women have a major impact on their own and their children's health [4, 5].

Lactating women are nutritionally vulnerable groups because this period places a high nutritional demand on the mother and leads to nutritional stress [6-8]. The diet consumed by the lactating mother will help to fulfill their own nutritional needs and prevents depletion of their body stores, and also enable them to produce enough milk for their infants and children [9-11]. Poor nutrition during lactation poses a significant threat to mothers and children's survival, growth, and development [12-14].

In lower and lower-middle income countries, undernutrition is responsible for a significant proportion of maternal and child morbidity and mortality [10]. Globally, around 9.4% of adult women were affected by undernutrition [15]. In African countries, the figure of underweight in reproductive-age women ranges from 5-20% [16, 17]. In Ethiopia, the national prevalence of undernutrition among reproductive-age women, as measured by BMI $<18.5 kg/m^2$, is 22.4%, while in the Afar region it is 39.1% [18]. Other mini-review done on lactating mothers also identified that; the prevalence of undernutrition is highest (50.6%) in the northern parts and lowest (17.4%) in the southern parts of Ethiopia [19].

Despite the number of effective and proven nutrition intervention programs in Ethiopia [20, 21], studies reported that a significant proportion of lactating mothers are undernourished [22-24]. Evidence-based, timely, and contextualized information on the nutritional status and related factors is vital to review those programs and strategies.

Even though there are a limited number of studies conducted to assess the nutritional status of lactating women in different regions of Ethiopia, there is no documented study done in the Afar region. Therefore, this study aimed to assess the nutritional status of lactating mothers and associated factors in the pastoral community, Afar region, northeast Ethiopia.

Methods and materials

Study design, setting, and period

A community-based cross-sectional study was conducted in the Abala district. It is one of the districts in zone two of the Afar region, located at the base of the eastern escarpment of the Ethiopian highlands about 942 kilometers northeast of Addis Ababa and 491 km far from the regional capital city, Samara. According to the projection of the 2007 national Census, the district has a total population of 43,372 with an area of 1,188.72 square kilometers. The district had one general hospital, four health centers, and eight functional health posts [25]. The study period was from January 5/2020 to February 10/2020.

Eligibility criteria

The source population was all lactating women whose children aged less than 24 months in the Abala district. Mothers were included if they lived in the district for six or more months. Mothers were considered to be excluded if they were seriously ill and physically unable to fit for anthropometric measurements, but no lactating mother was found with such cases. Additionally, women who were or who suspect pregnancy, according to maternal reports, were excluded from the study.

Sample size determination

The sample size for the first objective (For assessing the prevalence of undernutrition): The sample size was calculated using a single population proportion formula with a 95% confidence level, 5% margin of error, 25% estimated prevalence of undernutrition (chronic energy deficiency) in the study done in Tigray region [24], and by considering a non-response allowance of 10%. The calculated sample size for assessing the first objective was 317.

The sample size for the second objective (to determine factors associated with undernutrition): The sample size was determined using Epi info version 7 considering the assumptions of 95% CI, 80% power, the ratio of unexposed to exposed 1:1, and assuming different predictors from researches conducted in Ethiopia [26-28]. The predictor variable nutritional advice gives the largest sample size 370 (**Table 1**).

Table 1: Predictor variables selected for determining sample size for assessing factors associated with nutritional status of lactating mothers in Abala district, Afar, Ethiopia.

No	Significant predictors	Citation	CI	Power	Cases: control	Percent of exposure (%)		Samples size including 10% non-response rate		
						Cases	Controls	Cases	Controls	Total
1.	Marital status	[26]	95	80	1:1	62.00	14.93	20	20	40
2.	Nutrition advice	[27]	95	80	1:1	14.90	27.30	185	185	**370**
3.	Meal frequency	[28]	95	80	1:1	61.76	35.13	62	62	124

Therefore, the final sample size for this study, to determine both objectives, was taken as 370. This is a minimum representative sample for the study population.

Sampling procedure

The sampling procedure was a multi-stage sampling. The Abala district has 14 kebeles from which 5 kebeles were selected randomly. In each selected kebeles, the list of all households having lactating mothers was located (from the health extension worker's family folder). Then the sample size was proportionally allocated based on the total number of lactating women in each selected kebele. Finally, study participants in each selected kebeles were recruited from the list using a systematic sampling technique, every fixed interval. In households where there are more than one lactating women, a lottery method was used to select one participant (**Figure 1**).

Data collection procedure and quality control measures

A semi-structured questionnaire was developed through a critical review of relevant literature [6, 19, 22-24, 26, 29-34]. The questionnaire had the following parts, namely socio-demographic characteristics, maternal health care practice, maternal feeding practice, sanitation and hygiene-related factors, and anthropometric measurements. The research questionnaire was prepared in the English and translated into the local language. The questionnaire was pre-tested and validated on 10% of the sample size in the none-selected kebeles of the Abala district. Data was collected by direct face-to-face interviewing with lactating mothers and measuring anthropometry.

Six diploma-holder health professionals as data collectors and two master-holder public health professionals as supervisors were recruited. They were selected based on their fluency in the local language and they were trained on data collection techniques.

The dietary diversity of the lactating women was assessed using the food and agricultural organization of United States (FAO) women's dietary diversity score (WDDS) assessment scale. It was calculated by a simple count and summing-up of the number of food groups an individual respondent consumed over the preceding 24-hours recall period regardless of the portion size. The WDDS were calculated using the nine food groups (starchy staples, legumes and nuts, milk and milk products, dark green leafy vegetables, other vitamin-A rich fruits and vegetables, other fruits and vegetables, organ meat, meat and fish, and eggs). The study participants were asked whether they ate or not from each food group 24-hours preceding the survey. The cut-off point for the minimum dietary diversity score (MDDS) is the consumption of at least four or more food groups [35].

The anthropometric measurements such as weight, height, and mid-upper arm circumference (MUAC) of the lactating mother were performed according to the world health organization (WHO) standardized procedures. The weight of lactating mothers was measured using the United Nations International Children's Emergency Fund (UNICEF) Electronic Scale with a maximum capacity of 150 kg and an accuracy of 0.1 kg. Scales were calibrated and standardized every morning before the start of measuring the mother's weight, using a 5 kg standard weight. Mothers' weight was measured with light clothing and without shoes.

Maternal height was measured with standing position using a portable stadiometer to the nearest 0.1 cm, by allowing the mothers to take off their shoes and standing up straight placing their back against the wall. Maternal MUAC was measured using UNICEF measuring tape to the nearest 0.1 cm by placing WHO MUAC measuring tape loosely on the upper-middle arm between the acromion and olecranon process of the non-dominant hand.

Continuous supervision and follow-up of the data collectors were made to review and check for completeness and consistency of the collected data on daily basis. The collected data was handled and stored carefully and appropriately.

Data processing and analysis

The raw data was coded, cleaned, edited, and entered into Epi-data version 4.2 and transferred to statistical package for social sciences (SPSS) version 22 for analysis. The result of categorical variables was presented using frequency and percentage, bar graph and pie charts, and that of

continuous variables with a mean (SD). For continuous variables, normality was checked using graph (histogram) and statistics (Kolmogorov–Smirnov Test).

Bivariable and multivariable logistic regressions were used to assess the association of predictor variables with the outcome variable. Significance was determined using the adjusted and unadjusted odds ratio with 95% CI and P-value. All predictor variables that have an association with the outcome variable at bivariable analysis with the P value of $\leq$ 0.25 were selected and included in the multivariable logistic regression model. Variables with a P-value < 0.05 in multivariable analysis were declared as statistically and independently significant predictors of undernutrition among lactating mothers.

The final model was tested using Hosmer and Lemeshow's chi-square test p-value, and the P – value was 0.556, which showed that the model was the best fit. The percentage of the model that was accurately classified was 74% and the extent of multicollinearity was also assessed using standard error cut-off two, but there was no multicollinearity among studied variables.

Ethical consideration

The study protocol was approved by the Institutional Ethical Review Committee of Samara University; College of Health and Medical Science (ERC0053/2019). Written informed consent was obtained from lactating mothers, and confidentiality was also assured.

Operational definitions/Definition of terms

- **Maternal nutritional status**: - is measured using either BMI or MUAC. The mother was considered as
    - Undernourished: if her BMI < 18.5 Kg/m^2 and/or MUAC< 23cm
    - Not undernourished: if her BMI $\geq$ 18.5 kg/m^2 and/or MUAC $\geq$ 23 cm
- **Women dietary diversity score (WDDS):- is calculated using the** nine food groups' category [35, 36]. The mother was asked the type of food she ate 24-hours preceding the survey regardless of the portion size. WDDS is considered as
    - **Low**: if the mother consumed $\leq$ 3 food groups out of the nine food groups
    - **Moderate**: if the mother consumed 4-5 food groups out of the nine food groups
    - **High**: if the mother consumed $\geq$ 6 food groups out of the nine food groups
- **Minimum Meal frequency (MMF)**:- is the frequency or number of meals served by lactating mother 24-hours preceding the survey. The recommended energy intake during the

first 6 months of lactation is an additional 500 kilocalorie, which can be achieved by a dense extra meal per day [37, 38].

- **Meet:** ≥ 4 times
- **Not meet:** < 4 times

- **Lactating mother**: A mother who was breastfeeding her child at the time of the survey

Result

Socio-demographic characteristics of study participants

A total of 366 lactating mothers were included in this study, with a response rate of 98.9%. The mean (±SD) ages of study participants were 29.6(±7) years. The majority, 298(81.4%) and 311(85%) of them were rural residents and Afar in ethnicity, respectively. Three-fifth, 223(60.9%) of mothers were not attending formal education. The mean (±SD) of the study participant's family size was 5.6(+2.2), and the mean age of the index children was 11.5(±5.9) months (**Table 2**).

Table 2: Socio-demographic characteristics of study participants (n=366) in the Abala district, Afar region, northeast Ethiopia, 2020.

Variables	Category	Frequency (n)	Percentage (%)
Maternal age	15-24	107	29.2
	25-34	161	44.0
	35-49	98	26.8
	Mean (±SD)	29.6 ± 7.0	
Residence	Rural	298	81.4
Ethnicity	Afar	311	85.0
	Others*	55	15.0
Religion	Muslim	312	85.2
	Orthodox	54	14.8
Maternal marital status	Married	360	98.4
	Unmarried**	6	1.6
Maternal educational status	Not attended formal education	223	60.9
	Attended 1° education	106	29.0
	Attended 2° and above education	37	10.1
Maternal occupation	Pastoralist	168	45.9
	Housewife	120	32.8
	Merchant	50	13.7
	Employed***	28	7.6
Paternal educational status (n=360)	Not attended formal education	228	63.3
	Attend 1° education	60	16.7
	Attend 2° and above education	72	20.0

Paternal occupation (n=360)	Pastoralist	235	65.3
	Employed***	69	19.2
	Others ****	56	15.5
Family size	≤ 4	143	39.1
	5-8	181	49.4
	≥ 9	42	11.5
	Mean (±SD)	5.6(±2.2)	
Number of U-5 children within the household	One child	113	30.9
	Two or more children	253	69.1
Time taken to reach to Near-by Health Institution	<15 minute	173	47.3
	15-30 minute	188	51.3
	>30 minute	5	1.4
Decision maker of the household	Mostly father	252	68.9
	Mostly mother	63	17.2
	Jointly	51	13.9
Owing Television and/or Radio (yes)		60	16.4
Farming land ownership (yes)		106	29.0
Livestock ownership (yes)		299	81.7
Sex of the child	Male	176	48.1
Child age (in months)	0-5	73	19.9
	6-11	106	29.0
	12-23	187	51.1
	Mean (+SD)	11.5(±5.9)	

*Tigray, Amhara
**Single, divorced, widowed
***Employed in government and non-governmental organizations
****Merchant, daily laborer, farming

Maternal healthcare-related characteristics

Less than two-third, 229(62.6%) of mothers were attended antenatal care (ANC) follow-up. One hundred five (28.7%) of the study participants conceive their first pregnancy during their teenage. More than half, 198(54.1) of participants had less than 36 months of preceding birth interval. Nearly three-fourth, 265(72.4%) of the participants gave birth, to the index child, at home (**Table 3**).

Table 3: Maternal healthcare-related characteristics of study participants in the Abala district, Afar region, northeast Ethiopia, 2020.

Variables	Category	Frequency (n=366)	Percentage (%)
Antenatal care (ANC)	Yes	229	62.6
ANC frequency (n=229)	< 4 times	166	72.5
	≥ 4 times	63	27.5
Maternal age during first pregnancy	< 20 Years	105	28.7

	≥ 20 years	261	71.3
Gravidity	Primi-gravida	67	18.3
	2-4	169	46.2
	≥ 5	130	35.5
Birth interval	First child	67	18.3
	< 36 months	198	54.1
	≥ 36 months	101	27.6
Birth place	Home	265	72.4
	Institutional	101	27.6
Getting diseased in the past two months	Yes	81	22.1
Getting nutrition counseling	Yes	228	62.3
Maternal workload	Yes	78	21.3

Maternal nutritional status

Maternal nutritional status was assessed using both BMI and MUAC. According to BMI, 120(32.8%) of mothers were undernourished; with mild, moderate, and severe degrees of undernourishment occurred in 80(21.9%), 30(8.2%), and 10(2.7%) of respondents, respectively. But using MUAC, 122 (33.3%) of mothers were undernourished. The Pearson's correlation coefficient(r) between BMI and MUAC was 0.764 (P-value <0.001). The mean (±SD) score of mothers BMI, and MUAC were 20.2(±2.8) kg/m2, 23.9(±2.5) cm, respectively (**Table 4** and **Figure 2**).

Table 4: Nutritional status of study participants in the Abala district, Afar region, northeast Ethiopia, 2020.

Variables	Category	Frequency	Percentage (%)
Maternal BMI (kg/m^2)	<18.5	120	32.8
	18.5-24.9	230	62.8
	25-29.9	15	4.1
	≥ 30	1	0.3
	Mean (+SD)	20.2(±2.8)	
Degree of undernutrition (n=120)	Mild (BMI:17-18.49 kg/m^2)	80	21.9
	Moderate (BMI:16-16.69kg/m^2)	30	8.2
	Severe (BMI:<16kg/m^2)	10	2.7
Maternal MUAC (cm)	< 23	122	33.3
	≥23	244	66.7
	Mean (±SD)	23.9(±2.5)	

Maternal Feeding practice

Only 34(9.3%), and 114(31.1%) of mothers received at least one extra meal intake during pregnancy and lactation per day, respectively. Less than one-fourth, 88(24%) of mothers met the MDDS, while 120(32.8%) of them met the minimum meal frequency. Based on the 24-hours recall method, the mean (±SD) score of Maternal dietary diversity score and maternal meal frequency was 3.09(±0.875) and 3.33(±0.570), respectively.

Two-hundred seventy-eights (76%) of study participants had a low dietary diversity score (**Figure 3**). In the last 24-hours preceding the survey, all study participants consumed starchy staples, but only 16.9%, 3.8%, and 2.7% of them received dark green leafy vegetables, eggs, and organ meat, respectively (**Figure 4**).

Sanitation and hygiene-related characteristics

Three hundred thirty-seven (92.1%) of the study participants got water from protected sources. Less than half, 163(44.5%) of households had a latrine, from which 155(42.3) is functional at the time of the survey. Regarding type of latrine, majority 138(84.7%) is pit type, and the rest 25(15.3) is ventilated improved pit latrine (VIPL) type. Nearly three-fourth, 271(74%) of the study participants dispose of solid waste in open-field outside their compound, while 73(20%) and 22(6%) of the dispose in private pit, and open field in their compound.

All study participants, 366(100%), had the practice of hand-washing before preparing food and before feeding themselves. However, only 71(19.4%) of them used soap for hand-washing (**Figure 5).**

Factors associated with undernutrition

At binary logistic regression material age and education, paternal education and occupation, farming land ownership, maternal ANC follow-up, gravidity, birth interval, getting nutritional counseling, maternal dietary diversity score, and meal frequency were associated with maternal undernutrition at p-value<0.25. But **i**n the final multi-variable logistic regression model, maternal dietary diversity and birth interval were found statistically significantly associated (at the p-value < 0.05) with underweight among lactating mothers. Lactating mothers who didn’t meet the MDDS were more than five (AOR= 5.103; 95% CI: 2.128, 12.238) times more likely to be underweight than those who met the MDDS. Mothers with short birth intervals were more than four (AOR=4.800; 95% CI: 2.408, 9.567) time more likely to be undernourished (**Table 5**).

Table 5: Factors associated, at binary and multivariable logistic regression, with undernutrition among lactating mothers in the Abala district, Afar region, northeast Ethiopia				
**Variables / Category**	**Undernutrition (BMI<18.5kg/m^2)**		**COR(95% CI)**	**AOR(95% CI)**
	**Yes**	**No**		
**Maternal age (Years)**				
15-24	28	79	0.54(0.30, 0.97)	0.58(0.18, 1.88)
25-34	53	108	0.74(0.44, 1.25)	0.85(0.37, 1.95)
35-49	39	59	1	1
**Maternal education**				
Not educated	80	143	2.03(0.89, 4.65)	0.59(0.16, 2.15)
Attend primary school	32	74	1.57(0.65, 3.80)	0.56(0.17, 1.90)
Attend secondary & above	8	29	1	1
**Paternal education**				
Not educated	82	146	2.33(1.22, 4.43)	1.96(0.57, 6.76)
Attend primary school	21	39	2.23(1.01, 4.91)	1.69(0.58, 4.92)
Attend secondary & above	14	58	1	1
**Paternal occupation**				
Employed	15	54	1	1
Pastoralist	81	154	1.89(1.01, 3.56)	0.68(0.22, 2.10)
Others	21	35	2.16(0.98, 4.75)	1.68(0.63, 4.47)
**Farming-land ownership**				
Yes	23	83	1	1
No	97	163	2.15(1.27, 3.63)	1.77(0.98, 3.20)
**Maternal ANC follow-up**				
Yes	69	160	1	1
No	51	86	1.38(0.88, 2.15)	0.85(0.48, 1.50)
**Gravidity**				
Primi-gravida	16	51	1	1
2-4	53	116	1.46(0.76, 2.79)	0.48(0.05, 5.15)
$\geq$ 5	51	79	2.06(1.06, 3.99)	0.39(0.03, 4.69)
**Birth interval**				
First birth	16	51	1.80(0.82, 3.94)	1.02(0.10, 10.83)
< 36 month	89	109	4.68(2.53, 8.67)	**4.80(2.41, 9.57)***
$\geq$ 36 months	15	86	1	**1**
**Receiving nutrition counseling**				
Yes	66	162	1	1
No	54	84	1.58(1.01, 2.46)	1.29(0.75, 2.20)
**Maternal minimum dietary diversity score**				
Meet	10	78	1	**1**

Not meet	110	168	5.11(2.53, 10.29)	**5.10(2.13, 12.24)***
**Maternal minimum meal frequency**				
Meet	26	94	1	1
Not meet	94	152	2.24(1.35, 3.70)	1.31(0.70, 2.43)
*significant at P-value<0.001				

Discussion

This study found that the level of undernutrition among lactating mothers is 32.8% (95% CI: 28, 38). This finding was similar to a study conducted in Genta Afeshum woreda of rural Tigray which reported 33.6% [30]. The insignificant difference might be the data collection period for the later study was conducted during Ethiopian Orthodox lent fasting period which might affect dietary feeding practice and nutritional status of the mother, while the study collection period for this study was not on the fasting period.

Compared to the findings of this study, a lower prevalence of undernutrition among lactating mothers was reported in Offa district, Wolyita Zone 15.8% [26], Moyale district, Borena Zone 17.7% [33], Ambo district, West Shewa Zone 21.5% [32], Wombera woreda 25.4% [23], Arba mich Zuria district 26.1% [39], and Raya-Alamata district (Lowland 17.5%, highland 24.6%) [29]. It is also higher than the studies from Nigeria [31] and Zambia [40], in which 4.7% and 3.4% of lactating mothers were undernourished, respectively. The possible reason might be due to the different socio-demographic nature of the area; this study is in the pastoralist community while the other studies were more agrarian.

On the contrary, the finding of this study is lower than the findings from studies done in the Eastern zone of Tigray 38% [34], and Jimma, 40.6% [22]. The possible discrepancy might due to the difference in agro ecologic and cultural variation of the study areas. The other explanation for the observed difference might be the participants included in a study done in Jimma were beneficiaries of the home-based food production and child-centered counseling project.

Maternal dietary diversity score and birth interval were showed a statistically significant association with undernutrition among lactating mothers. Based on this study, lactating mothers who didn't meet the MDDS were more than five times more likely to be underweight as compared to those who met the MDDS. This is consistent with studies conducted in Jimma [22], Borena [33], and Raya-Alamata districts[29]. This can be explained by dietary diversity is a

proxy indicator of maternal nutrient adequacy, and this nutrient adequacy is essential for protein, lipid, and carbohydrate metabolism [41]. Maternal nutrient needs increase during lactation, and when these needs are not met, they may suffer from malnutrition. During lactation, the energy, protein, and other nutrients in breast milk come from a mother's diet or her body stores [42]. If lactating women do not get enough energy and nutrients they will be at risk of nutrient storage depletion and exacerbates undernutrition [43]. Monotonous diets full of starchy staples, low quantities of fruits and vegetables, and scant animal-source foods result in malnutrition [44, 45].

This study revealed that the probability of being undernutrition is more than four times among lactating mothers who had short birth intervals as compared to mothers who had a long duration of the birth interval. This finding is consistent with studies done in Nigeria [46, 47]. A birth interval of at least 36 months before couples deliver the next child is recommended for mothers and their children's health [48]. Lactation reduces the nutritional reserves of mothers, so longer inter-birth intervals allow for an increase in nutritional reserves [49]. The short birth interval could not give time to the mother to restore her nutritional status, and expose the mother to nutritional stress, morbidity, and mortality [50, 51].

In this study, even though the socio-demographic variables have a significant association at bivariate analysis, they were not significantly associated in the final multivariable model. But some studies [6, 23, 24, 26, 32] showed that family size, maternal education, maternal occupation, farming land ownership, and paternal education and occupation showed a significant association with undernutrition among lactating mothers. Also, contrary to other studies [23] maternal health care characteristics such as teenage pregnancy, ANC follow-up, place of delivery, postnatal care follow-up, maternal meal frequency, nutrition-related education, and maternal workloads had no statistically significant association with maternal undernutrition at multivariable logistic regression model. This difference is might be due to the difference in socio-demographic characteristics of the study areas. This study is conducted in a pastoralist community, while the other studies are mostly agrarian and with better access to health care services.

Strengths of the study

Using primary data, and being a community-based study, which will estimate the true prevalence of the problem.

Limitations of the study

Food consumption patterns might vary seasonally, and the results regarding dietary diversity score and meal frequency are limited to the specific season of the year in which the study was conducted. Those dietary assessments are also recorded using a 24-hour recall method; as a result, they might not reflect the usual intake of an individual. The other limitation of this study is failure to incorporate household food security and wealth index as factors associated with undernutrition which might have also introduced a residual confounding problem;

Conclusions

In this study, nearly one-third of lactating mothers were undernourished, which is higher as compared to other similar studies, and considered as a significant public health problem. Undernutrition was significantly associated with maternal dietary diversity and birth interval. For the prevention of undernutrition among lactating mothers health education on proper and adequate maternal dietary feeding practices, and proper family planning utilization during lactation should be emphasized.

Acknowledgments

We acknowledge the Afar regional and woreda health office heads for their valuable cooperation during the data collection. We would like to extend our gratitude to the data collectors and lactating mothers who participated in this study. Finally, thanks to Samara University for ethical approval and facilitation of this study.

Authors' contribution

Conceptualization: Getahun Fentaw Mulaw

Led data collection and investigation: Getahun Fentaw Mulaw and Kusse Urmale Mare

Formal Analysis and methodology: Getahun Fentaw Mulaw and Kusse Urmale Mare

Writing - original draft: Getahun Fentaw Mulaw

Writing – review and editing: Getahun Fentaw Mulaw and Kusse Urmale Mare, Etsay woldu Anbesu

References

1. Blossner M, De Onis M, Prüss-Üstün A. Malnutrition: quantifying the health impact at national and local levels: World Health Organization; 2005.

2. Unicef. Tracking Progress on Child and Maternal Nutrition: A Survival and Development Priority. eSocialSciences; 2015.

3. Meijers JM, Schols JM, Soeters PB, Halfens RJ. Defining malnutrition: mission or mission impossible? Nutrition. 2010;26(4):432-40.

4. World Health Organization. Progress in partnership: 2017 Progress report on the every woman every child global strategy for Women's, Children's and adolescents' health. World Health Organization; 2017.

5. Black R, Allen L, Bhutta Z, Caulfield L, Onis. Maternal and child undernutrition 1-Maternal and child undernutrition: global and regional exposures and health consequences. Lancet. 2008.

6. Hundera TD, Gemede HF, Wirtu D, Kenie DN. Nutritional status and associated factors among lactating mothers in Nekemte Referral Hospital and Health Centers, Ethiopia. Int J Nutr Food Sci. 2015;4(2):216-22.

7. Walker SP. Nutritional issues for women in developing countries. Proceedings of the Nutrition Society. 1997;56(1B):345-56.

8. Khan Y, Bhutta ZA. Nutritional deficiencies in the developing world: current status and opportunities for intervention. Pediatric Clinics. 2010;57(6):1409-41.

9. Allen LH. B vitamins in breast milk: relative importance of maternal status and intake, and effects on infant status and function. Advances in nutrition. 2012;3(3):362-9.

10. Black RE, Allen LH, Bhutta ZA, Caulfield LE, De Onis M, Ezzati M, et al. Maternal and child undernutrition: global and regional exposures and health consequences. The lancet. 2008;371(9608):243-60.

11. Nakamori M, Ninh NX, Isomura H, Yoshiike N, Hien VTT, Nhug BT, et al. Nutritional status of lactating mothers and their breast milk concentration of iron, zinc and copper in rural Vietnam. Journal of nutritional science and vitaminology. 2009;55(4):338-45.

12. Westspot C. Nutrition in the first 1,000 days: State of the world's mothers 2012. Westport, CT: Save the Children. 2012. ISBN 1-888393-24; 2012.

13. Negash C, Whiting SJ, Henry CJ, Belachew T, Hailemariam TG. Association between maternal and child nutritional status in Hula, rural Southern Ethiopia: A cross sectional study. PloS one. 2015;10(11):e0142301.

14. Ahmed T, Hossain M, Sanin KI. Global burden of maternal and child undernutrition and micronutrient deficiencies. Annals of Nutrition and Metabolism. 2012;61(Suppl. 1):8-17.

15. UNICEF/World Health Organization (WHO)/World Bank Group. Global Nutrition Repot; The burden of malnutrition: Exploring global and regional trends in malnutrition in all its forms; 2020 :

Available from: https://globalnutritionreport.org/reports/global-nutrition-report-2018/burden-malnutrition/

16. Huffman SL, Zehner E, Harvey P, Martin L, Piwoz E, Ndure K, et al. Essential health sector actions to improve maternal nutrition in Africa. LINKAGES Project, Academy for Educational Development. 2001.

17. Achadi E, Ahuja A, Bendech MA, Bhutta ZA, De-Regil LM, Fanzo J, et al. Global nutrition report 2016: From promise to impact: Ending malnutrition by 2030: International Food Policy Research Institute; 2016.

18. Central Statistical Agency (Federal Democratic Republic of Ethiopia). Ethiopian Demographic and Health Survey 2016.

19. Sserwanja Q, Kawuki J. Prevalence of underweight and associated factors among lactating women in Ethiopia: a mini-review. Journal of Advances in Medicine and Medical Research. 2020:1-9.

20. Federal Ministry of Health Adis Ababa (Federal Democratic Republic of Ethiopia). Health Sector Transformation Plan 2015/16–2019/20: Available from: https://www.globalfinancingfacility.org/ethiopia-health-sector-transformation-plan-201516-201920.

21. Wang H, Tesfaye R, NV Ramana G, Chekagn CT. Ethiopia health extension program: an institutionalized community approach for universal health coverage: The World Bank; 2016.

22. Alemayehu M, Argaw A, Mariam AG. Factors associated with malnutrition among lactating women in Subsistence farming households from Dedo and Seqa-Chekorsa districts, Jimma zone, 2014. Developing Country Studies. 2015;5(21):117-8.

23. Berihun S, Kassa GM, Teshome M. Factors associated with underweight among lactating women in Womberma woreda, Northwest Ethiopia; a cross-sectional study. BMC nutrition. 2017;3(1):1-7.

24. Haileslassie K, Mulugeta A, Girma M. Feeding practices, nutritional status and associated factors of lactating women in Samre Woreda, South Eastern Zone of Tigray, Ethiopia. Nutrition journal. 2013;12(1):1-11.

25. Health Department. Abala woreda (Afar region, Ethiopia) helth bureaue report. Juanuary 2020. .

26. Julla BW, Haile A, Ayana G, Eshetu S, Kuche D, Asefa T. Chronic Energy Deficiency and Associated Factors among Lactating Mothers (15-49 years old) in Offa Woreda, Wolayita Zone, SNNPRs, Ethiopia. World Scientific Research. 2018;5(1):13-23.

27. Abeya S, Biru K, Jima A. Factors Associated with Underweight among Lactating Mothers in Adama District, Oromia Region, Ethiopia. J Orthop Bone Res 1: 101 Abstract Keywords: Lactating Women. 2018.

28. Abraham S, Miruts G, Shumye A. Magnitude of chronic energy deficiency and its associated factors among women of reproductive age in the Kunama population, Tigray, Ethiopia, in 2014. BMC nutrition. 2015;1(1):1-9.

29. Sitotaw IK, Hailesslasie K, Adama Y. Comparison of nutritional status and associated factors of lactating women between lowland and highland communities of District Raya, Alamata, Southern Tigiray, Ethiopia. BMC nutrition. 2017;3(1):1-12.

30. Desalegn BB, Lambert C, Riedel S, Negese T, Biesalski HK. Ethiopian orthodox fasting and lactating mothers: Longitudinal study on dietary pattern and nutritional status in rural tigray, Ethiopia. International journal of environmental research and public health. 2018;15(8):1767.

31. Sanusi R, Falana O. The nutritional status of mothers practicing breast feeding in Ibadan, Nigeria. African Journal of Biomedical Research. 2009;12(2):107-12.

32. Zerihun E, Egata G, Mesfin F. Under nutrition and its associated factors among lactating mothers in rural Ambo district, West Shewa zone, Oromia region, Ethiopia. East African Journal of Health and Biomedical Sciences. 2016;1(1):39-48.

33. Bekele H, Jima GH, Regesu AH. Undernutrition and associated factors among lactating women: Community-based cross-sectional study in Moyale District, Borena Zone, Southern Ethiopia. Advances in Public Health. 2020;2020.

34. Abera SF, Kantelhardt EJ, Bezabih AM, Tsadik M, Lauvai J, Ejeta G, et al. What factors are associated with maternal undernutrition in eastern zone of Tigray, Ethiopia? Evidence for nutritional well-being of lactating mothers. BMC public health. 2020;20(1):1-12.

35. Kennedy G, Ballard T, Dop MC. Guidelines for measuring household and individual dietary diversity: Food and Agriculture Organization of the United Nations; 2011.

36. FAO and FHI 360. Minimum dietary diversity for women: a guide for measurement. Rome: FAO. 2016;82.

37. Be CNNDL, Alone MBUDI. 9 Meeting Maternal Nutrient Needs During Lactation.

38. Picciano MF. Pregnancy and lactation: physiological adjustments, nutritional requirements and the role of dietary supplements. The Journal of nutrition. 2003;133(6):1997S-2002S.

39. Tikuye HH, Gebremedhin S, Mesfin A, Whiting S. Prevalence and factors associated with undernutrition among exclusively breastfeeding women in Arba Minch Zuria District, Southern Ethiopia: A cross-sectional community-based study. Ethiopian journal of health sciences. 2019;29(1).

40. Kaliwile C, Michelo C, Titcomb TJ, Moursi M, Donahue Angel M, Reinberg C, et al. Dietary intake patterns among lactating and non-lactating women of reproductive age in rural Zambia. Nutrients. 2019;11(2):288.

41. Arimond M, Wiesmann D, Becquey E, Carriquiry A, Daniels MC, Deitchler M, et al. Simple food group diversity indicators predict micronutrient adequacy of women's diets in 5 diverse, resource-poor settings. The Journal of nutrition. 2010;140(11):2059S-69S.

42. Dewey KG, Cohen RJ, Brown KH, Rivera LL. Effects of exclusive breastfeeding for four versus six months on maternal nutritional status and infant motor development: results of two randomized trials in Honduras. The Journal of nutrition. 2001;131(2):262-7.

43. Dewey KG. Energy and protein requirements during lactation. Annual review of nutrition. 1997;17(1):19-36.

44. Grech AM, Alders R, Darnton-Hill I, Bagnol B, Hikeezi D, O'Leary F. Nutrition Knowledge, Attitudes, and Dietary Intake of Women of Reproductive Age in Bundabunda Ward, Zambia. Clinical Journal of Nutrition and Dietetics. 2018;1(2):1-12.

45. De Bruyn J, Bagnol B, Darnton-Hill I, Maulaga W, Thomson PC, Alders R. Characterising infant and young child feeding practices and the consumption of poultry products in rural Tanzania: A mixed methods approach. Maternal & Child Nutrition. 2018;14(2):e12550.

46. Adebowale S, Adepoju O, Fagbamigbe F. Child spacing and parity progression: Implication for maternal nutritional status among women in Ekiti communities, Southwestern Nigeria. Pakistan Journal of Nutrition. 2011;10(5):485-91.

47. Kathryn G, Roberta J. Birth Spacing Literature: Maternal and Child Nutrition Out Comes. Program in international nutrition, university of Califonia, Davis. 2004.

48. World Health Organization. Report of a WHO technical consultation on birth spacing: Geneva, Switzerland 13-15 June 2005. World Health Organization; 2007.

49. King JC. The risk of maternal nutritional depletion and poor outcomes increases in early or closely spaced pregnancies. The Journal of nutrition. 2003;133(5):1732S-6S.

50. Rutstein S, editor Effect of birth intervals on mortality and health: multivariate cross country analyses. Conference on Optimal Birth Spacing for Central America, Antigua, Guatemala; 2003.

51. Brockman S, Stout I, Marsh K. The Public Health Impact of Optimal Birth Spacing: New Research from Latin America and the Caribbean. 2003.

Figure Click here to access/download;Figure;Figures.docx

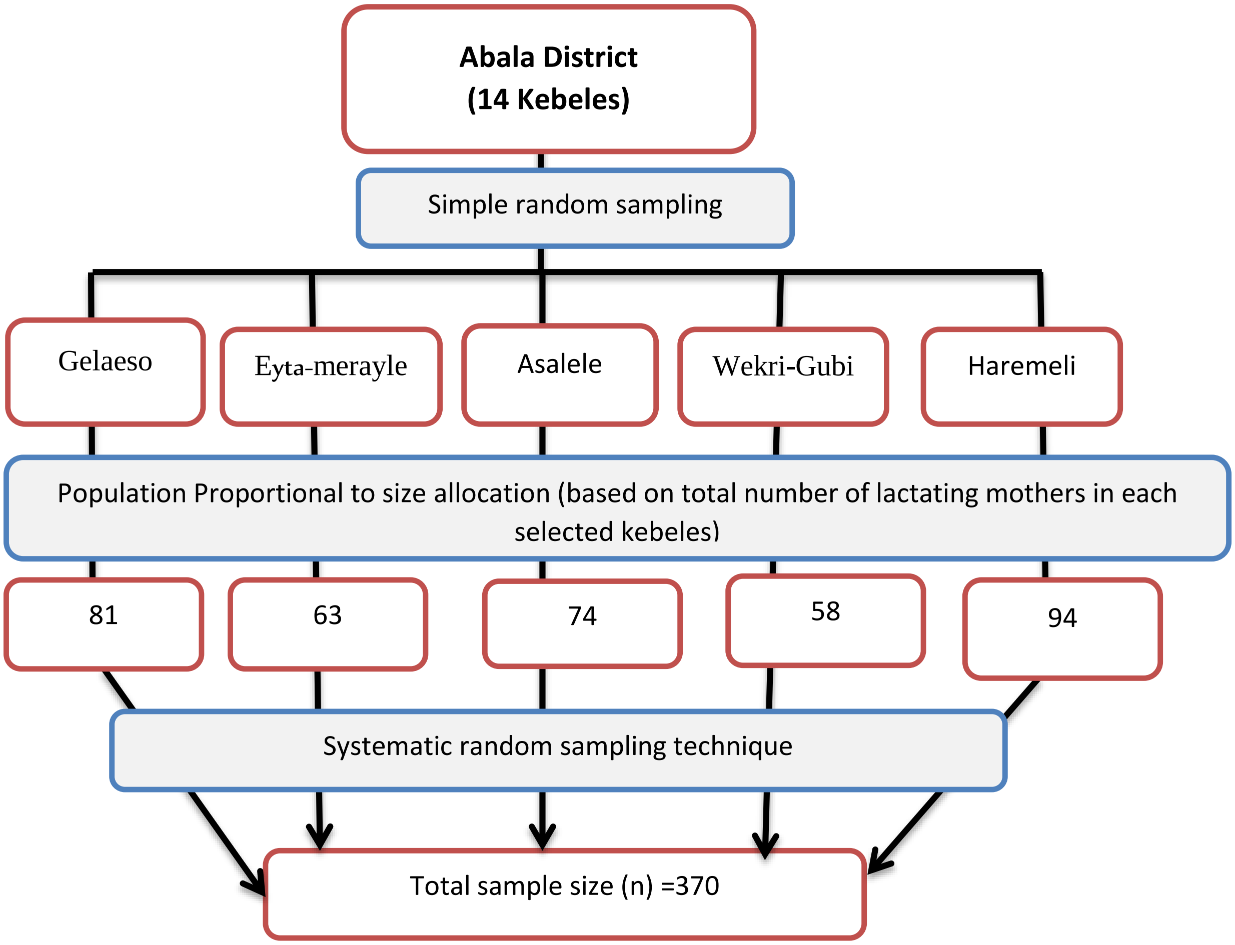

Figure 1: Schematic representation of sampling methods and procedures to select study participants, in Abala district, Afar region, northeast Ethiopia, 2020.

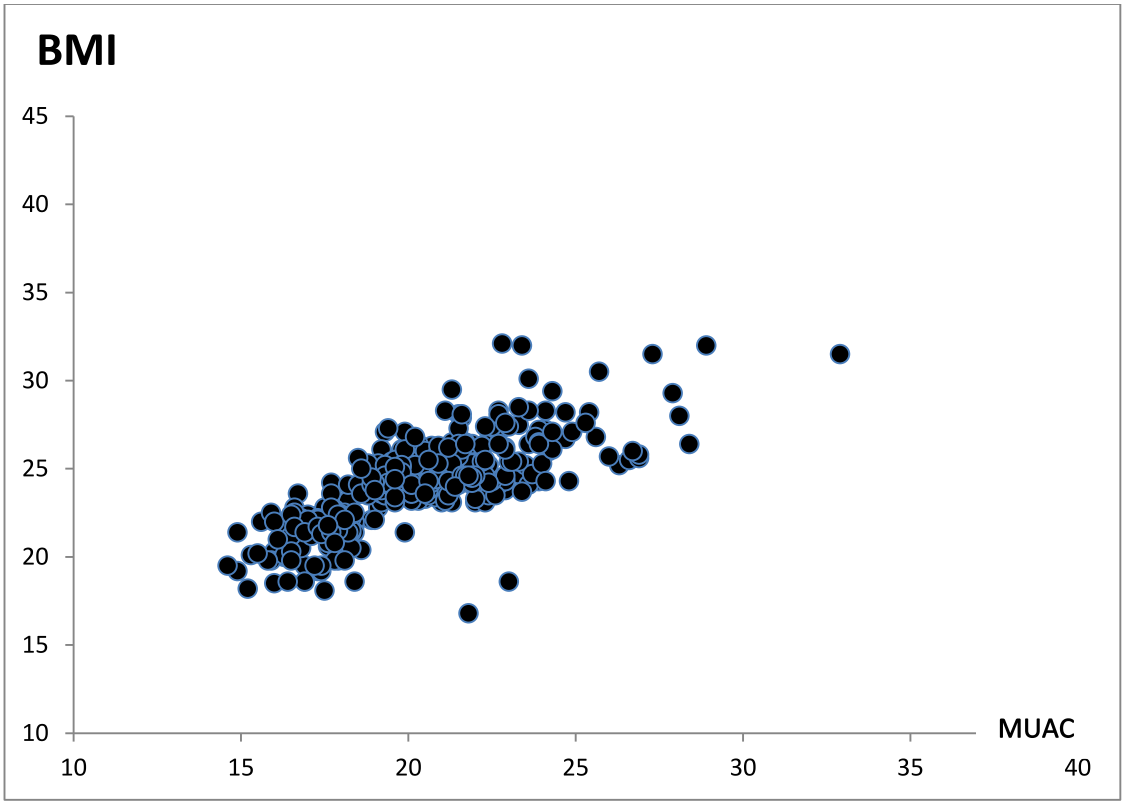

Figure 2: Scatter plot showing the relation between maternal BMI and maternal MUAC (r = 0.764), of study participants in Abala district, Afar region, northeast Ethiopia, 2020.

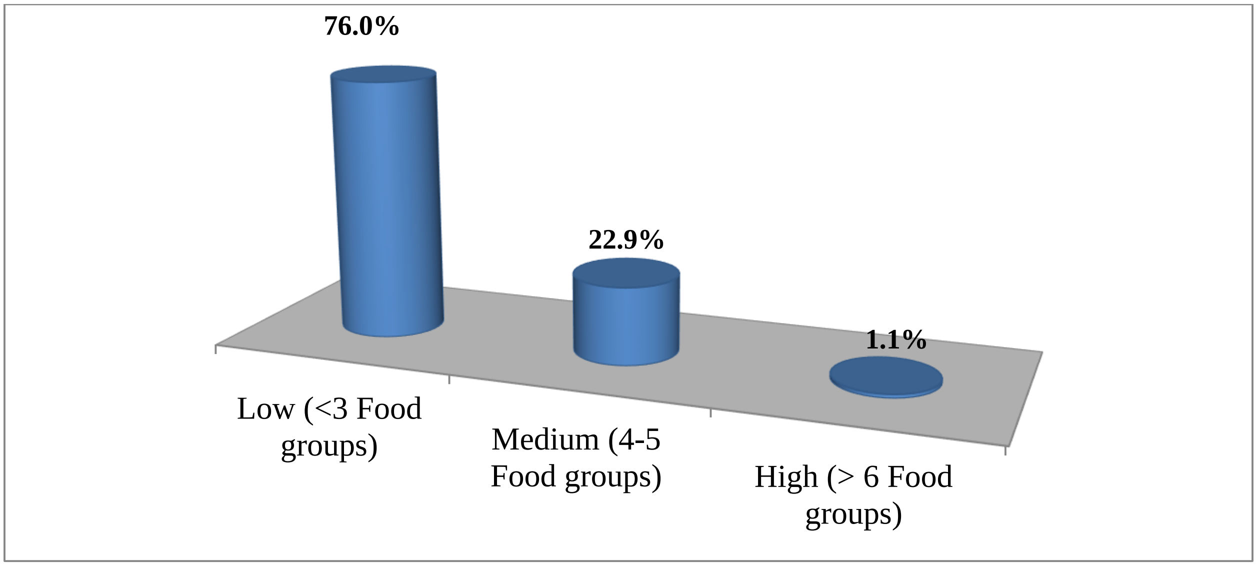

Figure 3: Level of dietary diversity score of study participants (n=366) in Abala district, Afar region, Northeast Ethiopia, 2020.

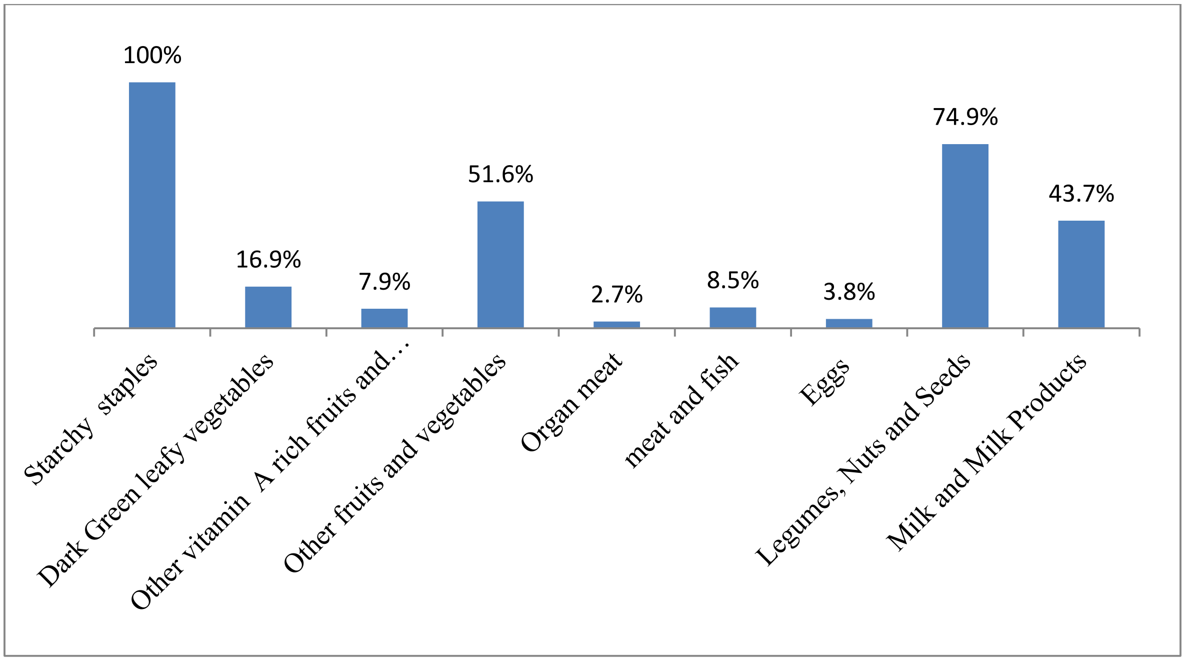

Figure 4: Pattern of food group consumption of study participants (n=366) in Abala district, Afar region, Northeast Ethiopia, 2020.

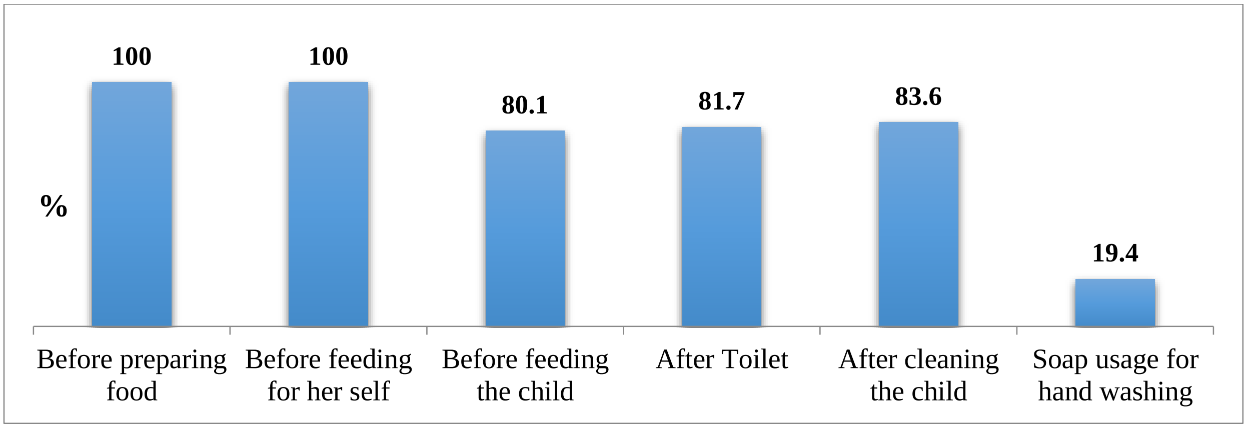

Figure 5: Proportion of hand-washing practice of study participants (n=366) in Abala district, Afar region, Northeast Ethiopia, 2020.

Click here to access/download
Supporting Information
S1 File.docx

Click here to access/download
Supporting Information
S1 Data.sav

Nearly One-Third of Lactating Mothers are Suffering from Undernutrition in Pastoral Community, Afar Region, Ethiopia: Community-Based Cross-Sectional Study

Getahun Fentaw Mulaw*[1], Kusse Urmale Mare[2], Etsay woldu Anbesu[3]

[1]School of Public Health, College of Health Sciences, Woldia University, Woldia, Amhara region, Ethiopia

[2]Nursing Department, College of Health Sciences, Samara University, Samara, Afar region, Ethiopia

[3]Public Health Department, College of Health Sciences, Samara University, Samara, Afar region, Ethiopia

***Corresponding author:** *GFM*; Email:gechfentaw1014@gmail.com; ORCID: https://orcid.org/0000-0002-4173-3759)

Abstract

Background: - Undernutrition is responsible for a significant proportion of maternal and child morbidity and mortality. Lactating women are nutritionally vulnerable groups because this period places a high nutritional demand on the mother and leads to nutritional stress. Poor nutrition during lactation has a significant negative consequence to mothers and children's survival, growth, and development. Therefore this study aimed to assess the nutritional status of lactating mothers and associated factors in pastoral community, Afar region, Ethiopia.

Methods: - A community-based cross-sectional study was conducted from January 5/2020 to February 10/2020, in the Abala district. The data were collected from a sample of 366 lactating mothers whose children aged less than 24 months. Data was collected through face-to-face interviews and anthropometric measurements. Study participants were recruited using a systematic sampling technique. Anthropometric measurements (both body mass index and mid-upper arm circumference) were taken from each mother using calibrated equipment and standardized techniques. Data were entered into Epi-data version 4.2 and exported to SPSS version 22 for analysis. Predictor variables with a P-value < 0.25 at bivariable analysis were candidates for the final model. Statistical significance was declared at P-value of < 0.05 in the multivariable logistic regression.

Result: - This study showed that 120(32.8%) and 122(33.3%) surveyed mothers were undernourished using a cut-off body mass index <18.5 kg/m^2 and mid-upper arm circumference <23 Centimeter, respectively. Lactating mothers who didn't meet the minimum dietary diversity score were more than five (Adjusted odds ratio (AOR) = 5.103; 95% confidence interval (CI): 2.128, 12.238) times more likely to be undernourished than those who met the minimum dietary diversity score. Mothers with short birth intervals were also more than four (AOR=4.800; 95% CI: 2.408, 9.567) time more likely to be undernourished.

Conclusion: - Nearly one-third of lactating mothers were undernourished. Undernutrition among lactating mothers was significantly associated with maternal dietary diversity score and birth interval. Health education on proper and adequate maternal dietary feeding practices, and proper family planning utilization during lactation should be emphasized.

Keywords: - Undernutrition, lactating mothers, Abala district, Pastoralist, Afar, Ethiopia

Background

Malnutrition is one of the most devastating problems worldwide and can be manifested as both undernutrition and overnutrition. Undernutrition can be divided into protein-energy malnutrition and micronutrient deficiencies [1]. Undernutrition is the outcome of insufficient food intake, inadequate care, and infectious diseases [2, 3]. Malnutrition among reproductive-age women have a major impact on their own and their children's health [4, 5].

Lactating women are nutritionally vulnerable groups because this period places a high nutritional demand on the mother and leads to nutritional stress [6-8]. The diet consumed by the lactating mother will help to fulfill their own nutritional needs and prevents depletion of their body stores, and also enable them to produce enough milk for their infants and children [9-11]. Poor nutrition during lactation poses a significant threat to mothers and children's survival, growth, and development [12-14].

In lower and lower-middle income countries, undernutrition is responsible for a significant proportion of maternal and child morbidity and mortality [10]. Globally, around 9.4% of adult women were affected by undernutrition [15]. In African countries, the figure of underweight in reproductive-age women ranges from 5-20% [16, 17]. In Ethiopia, the national prevalence of undernutrition among reproductive-age women, as measured by BMI $<18.5 kg/m^2$, is 22.4%, while in the Afar region it is 39.1% [18]. Other mini-review done on lactating mothers also identified that; the prevalence of undernutrition is highest (50.6%) in the northern parts and lowest (17.4%) in the southern parts of Ethiopia [19].

Despite the number of effective and proven nutrition intervention programs in Ethiopia [20, 21], studies reported that a significant proportion of lactating mothers are undernourished [22-24]. Evidence-based, timely, and contextualized information on the nutritional status and related factors is vital to review those programs and strategies.

Even though there are a limited number of studies conducted to assess the nutritional status of lactating women in different regions of Ethiopia, there is no documented study done in the Afar region. Therefore, this study aimed to assess the nutritional status of lactating mothers and associated factors in the pastoral community, Afar region, northeast Ethiopia.

Methods and materials

Study design, setting, and period

A community-based cross-sectional study was conducted in the Abala district. It is one of the districts in zone two of the Afar region, located at the base of the eastern escarpment of the Ethiopian highlands about 942 kilometers northeast of Addis Ababa and 491 km far from the regional capital city, Samara. According to the projection of the 2007 national Census, the district has a total population of 43,372 with an area of 1,188.72 square kilometers. The district had one general hospital, four health centers, and eight functional health posts [25]. The study period was from January 5/2020 to February 10/2020.

Eligibility criteria

The source population was all lactating women whose children aged less than 24 months in the Abala district. Mothers were included if they lived in the district for six or more months. Mothers were considered to be excluded if they were seriously ill and physically unable to fit for anthropometric measurements, but no lactating mother was found with such cases. Additionally, women who were or who suspect pregnancy, according to maternal reports, were excluded from the study.

Sample size determination

The sample size for the first objective (For assessing the prevalence of undernutrition): The sample size was calculated using a single population proportion formula with a 95% confidence level, 5% margin of error, 25% estimated prevalence of undernutrition (chronic energy deficiency) in the study done in Tigray region [24], and by considering a non-response allowance of 10%. The calculated sample size for assessing the first objective was 317.

The sample size for the second objective (to determine factors associated with undernutrition): The sample size was determined using Epi info version 7 considering the assumptions of 95% CI, 80% power, the ratio of unexposed to exposed 1:1, and assuming different predictors from researches conducted in Ethiopia [26-28]. The predictor variable nutritional advice gives the largest sample size 370 (**Table 1**).

Table 1: Predictor variables selected for determining sample size for assessing factors associated with nutritional status of lactating mothers in Abala district, Afar, Ethiopia.

No	Significant predictors	Citation	CI	Power	Cases: control	Percent of exposure (%)		Samples size including 10% non-response rate		
						Cases	Controls	Cases	Controls	Total
1.	Marital status	[26]	95	80	1:1	62.00	14.93	20	20	40
2.	Nutrition advice	[27]	95	80	1:1	14.90	27.30	185	185	**370**
3.	Meal frequency	[28]	95	80	1:1	61.76	35.13	62	62	124

Therefore, the final sample size for this study, to determine both objectives, was taken as 370. This is a minimum representative sample for the study population.

Sampling procedure

The sampling procedure was a multi-stage sampling. The Abala district has 14 kebeles from which 5 kebeles were selected randomly. In each selected kebeles, the list of all households having lactating mothers was located (from the health extension worker's family folder). Then the sample size was proportionally allocated based on the total number of lactating women in each selected kebele. Finally, study participants in each selected kebeles were recruited from the list using a systematic sampling technique, every fixed interval. In households where there are more than one lactating women, a lottery method was used to select one participant (**Figure 1**).

Data collection procedure and quality control measures

A semi-structured questionnaire was developed through a critical review of relevant literature [6, 19, 22-24, 26, 29-34]. The questionnaire had the following parts, namely socio-demographic characteristics, maternal health care practice, maternal feeding practice, sanitation and hygiene-related factors, and anthropometric measurements. The research questionnaire was prepared in the English and translated into the local language. The questionnaire was pre-tested and validated on 10% of the sample size in the none-selected kebeles of the Abala district. Data was collected by direct face-to-face interviewing with lactating mothers and measuring anthropometry.

Six diploma-holder health professionals as data collectors and two master-holder public health professionals as supervisors were recruited. They were selected based on their fluency in the local language and they were trained on data collection techniques.

The dietary diversity of the lactating women was assessed using the food and agricultural organization of United States (FAO) women's dietary diversity score (WDDS) assessment scale. It was calculated by a simple count and summing-up of the number of food groups an individual respondent consumed over the preceding 24-hours recall period regardless of the portion size. The WDDS were calculated using the nine food groups (starchy staples, legumes and nuts, milk and milk products, dark green leafy vegetables, other vitamin-A rich fruits and vegetables, other fruits and vegetables, organ meat, meat and fish, and eggs). The study participants were asked whether they ate or not from each food group 24-hours preceding the survey. The cut-off point for the minimum dietary diversity score (MDDS) is the consumption of at least four or more food groups [35].

The anthropometric measurements such as weight, height, and mid-upper arm circumference (MUAC) of the lactating mother were performed according to the world health organization (WHO) standardized procedures. The weight of lactating mothers was measured using the United Nations International Children's Emergency Fund (UNICEF) Electronic Scale with a maximum capacity of 150 kg and an accuracy of 0.1 kg. Scales were calibrated and standardized every morning before the start of measuring the mother's weight, using a 5 kg standard weight. Mothers' weight was measured with light clothing and without shoes.

Maternal height was measured with standing position using a portable stadiometer to the nearest 0.1 cm, by allowing the mothers to take off their shoes and standing up straight placing their back against the wall. Maternal MUAC was measured using UNICEF measuring tape to the nearest 0.1 cm by placing WHO MUAC measuring tape loosely on the upper-middle arm between the acromion and olecranon process of the non-dominant hand.

Continuous supervision and follow-up of the data collectors were made to review and check for completeness and consistency of the collected data on daily basis. The collected data was handled and stored carefully and appropriately.

Data processing and analysis

The raw data was coded, cleaned, edited, and entered into Epi-data version 4.2 and transferred to statistical package for social sciences (SPSS) version 22 for analysis. The result of categorical variables was presented using frequency and percentage, bar graph and pie charts, and that of

continuous variables with a mean (SD). For continuous variables, normality was checked using graph (histogram) and statistics (Kolmogorov–Smirnov Test).

Bivariable and multivariable logistic regressions were used to assess the association of predictor variables with the outcome variable. Significance was determined using the adjusted and unadjusted odds ratio with 95% CI and P-value. All predictor variables that have an association with the outcome variable at bivariable analysis with the P value of $\leq$ 0.25 were selected and included in the multivariable logistic regression model. Variables with a P-value < 0.05 in multivariable analysis were declared as statistically and independently significant predictors of undernutrition among lactating mothers.

The final model was tested using Hosmer and Lemeshow's chi-square test p-value, and the P – value was 0.556, which showed that the model was the best fit. The percentage of the model that was accurately classified was 74% and the extent of multicollinearity was also assessed using standard error cut-off two, but there was no multicollinearity among studied variables.

Ethical consideration

The study protocol was approved by the Institutional Ethical Review Committee of Samara University; College of Health and Medical Science (ERC0053/2019). Written informed consent was obtained from lactating mothers, and confidentiality was also assured.

Operational definitions/Definition of terms

- **Maternal nutritional status**: - is measured using either BMI or MUAC. The mother was considered as
  - Undernourished: if her BMI < 18.5 Kg/m^2 and/or MUAC< 23cm
  - Not undernourished: if her BMI $\geq$ 18.5 kg/m^2 and/or MUAC $\geq$ 23 cm
- **Women dietary diversity score (WDDS):- is calculated using the** nine food groups' category [35, 36]. The mother was asked the type of food she ate 24-hours preceding the survey regardless of the portion size. WDDS is considered as
  - **Low**: if the mother consumed $\leq$ 3 food groups out of the nine food groups
  - **Moderate**: if the mother consumed 4-5 food groups out of the nine food groups
  - **High**: if the mother consumed $\geq$ 6 food groups out of the nine food groups
- **Minimum Meal frequency (MMF)**:- is the frequency or number of meals served by lactating mother 24-hours preceding the survey. The recommended energy intake during the

first 6 months of lactation is an additional 500 kilocalorie, which can be achieved by a dense extra meal per day [37, 38].

  - **Meet:** ≥ 4 times
  - **Not meet:** < 4 times

- **Lactating mother**: A mother who was breastfeeding her child at the time of the survey

Result

Socio-demographic characteristics of study participants

A total of 366 lactating mothers were included in this study, with a response rate of 98.9%. The mean (±SD) ages of study participants were 29.6(±7) years. The majority, 298(81.4%) and 311(85%) of them were rural residents and Afar in ethnicity, respectively. Three-fifth, 223(60.9%) of mothers were not attending formal education. The mean (±SD) of the study participant's family size was 5.6(+2.2), and the mean age of the index children was 11.5(±5.9) months (**Table 2**).

Table 2: Socio-demographic characteristics of study participants (n=366) in the Abala district, Afar region, northeast Ethiopia, 2020.

Variables	Category	Frequency (n)	Percentage (%)
Maternal age	15-24	107	29.2
	25-34	161	44.0
	35-49	98	26.8
	Mean (±SD)	29.6 ± 7.0	
Residence	Rural	298	81.4
Ethnicity	Afar	311	85.0
	Others*	55	15.0
Religion	Muslim	312	85.2
	Orthodox	54	14.8
Maternal marital status	Married	360	98.4
	Unmarried**	6	1.6
Maternal educational status	Not attended formal education	223	60.9
	Attended 1° education	106	29.0
	Attended 2° and above education	37	10.1
Maternal occupation	Pastoralist	168	45.9
	Housewife	120	32.8
	Merchant	50	13.7
	Employed***	28	7.6
Paternal educational status (n=360)	Not attended formal education	228	63.3
	Attend 1° education	60	16.7
	Attend 2° and above education	72	20.0

Paternal occupation (n=360)	Pastoralist	235	65.3
	Employed***	69	19.2
	Others ****	56	15.5
Family size	< 4	143	39.1
	5-8	181	49.4
	> 9	42	11.5
	Mean (+SD)	5.6(+2.2)	
Number of U-5 children within the household	One child	113	30.9
	Two or more children	253	69.1
Time taken to reach to Near-by Health Institution	<15 minute	173	47.3
	15-30 minute	188	51.3
	>30 minute	5	1.4
Decision maker of the household	Mostly father	252	68.9
	Mostly mother	63	17.2
	Jointly	51	13.9
Owing Television and/or Radio (yes)		60	16.4
Farming land ownership (yes)		106	29.0
Livestock ownership (yes)		299	81.7
Sex of the child	Male	176	48.1
Child age (in months)	0-5	73	19.9
	6-11	106	29.0
	12-23	187	51.1
	Mean (+SD)	11.5(+5.9)	

*Tigray, Amhara
**Single, divorced, widowed
***Employed in government and non-governmental organizations
****Merchant, daily laborer, farming

Maternal healthcare-related characteristics

Less than two-third, 229(62.6%) of mothers were attended antenatal care (ANC) follow-up. One hundred five (28.7%) of the study participants conceive their first pregnancy during their teenage. More than half, 198(54.1) of participants had less than 36 months of preceding birth interval. Nearly three-fourth, 265(72.4%) of the participants gave birth, to the index child, at home (**Table 3**).

Table 3: Maternal healthcare-related characteristics of study participants in the Abala district, Afar region, northeast Ethiopia, 2020.

Variables	Category	Frequency (n=366)	Percentage (%)
Antenatal care (ANC)	Yes	229	62.6
ANC frequency (n=229)	< 4 times	166	72.5
	> 4 times	63	27.5
Maternal age during first pregnancy	< 20 Years	105	28.7

	≥ 20 years	261	71.3
Gravidity	Primi-gravida	67	18.3
	2-4	169	46.2
	≥ 5	130	35.5
Birth interval	First child	67	18.3
	< 36 months	198	54.1
	≥ 36 months	101	27.6
Birth place	Home	265	72.4
	Institutional	101	27.6
Getting diseased in the past two months	Yes	81	22.1
Getting nutrition counseling	Yes	228	62.3
Maternal workload	Yes	78	21.3

Maternal nutritional status

Maternal nutritional status was assessed using both BMI and MUAC. According to BMI, 120(32.8%) of mothers were undernourished; with mild, moderate, and severe degrees of undernourishment occurred in 80(21.9%), 30(8.2%), and 10(2.7%) of respondents, respectively. But using MUAC, 122 (33.3%) of mothers were undernourished. The Pearson's correlation coefficient(r) between BMI and MUAC was 0.764 (P-value <0.001). The mean (±SD) score of mothers BMI, and MUAC were 20.2(±2.8) kg/m2, 23.9(±2.5) cm, respectively (**Table 4** and **Figure 2**).

Table 4: Nutritional status of study participants in the Abala district, Afar region, northeast Ethiopia, 2020.

Variables	Category	Frequency	Percentage (%)
Maternal BMI (kg/m^2)	<18.5	120	32.8
	18.5-24.9	230	62.8
	25-29.9	15	4.1
	≥ 30	1	0.3
	Mean (+SD)	20.2(±2.8)	
Degree of undernutrition (n=120)	Mild (BMI:17-18.49 kg/m^2)	80	21.9
	Moderate (BMI:16-16.69kg/m^2)	30	8.2
	Severe (BMI:<16kg/m^2)	10	2.7
Maternal MUAC (cm)	< 23	122	33.3
	≥23	244	66.7
	Mean (±SD)	23.9(±2.5)	

Maternal Feeding practice

Only 34(9.3%), and 114(31.1%) of mothers received at least one extra meal intake during pregnancy and lactation per day, respectively. Less than one-fourth, 88(24%) of mothers met the MDDS, while 120(32.8%) of them met the minimum meal frequency. Based on the 24-hours recall method, the mean (±SD) score of Maternal dietary diversity score and maternal meal frequency was 3.09(±0.875) and 3.33(±0.570), respectively.

Two-hundred seventy-eights (76%) of study participants had a low dietary diversity score (**Figure 3**). In the last 24-hours preceding the survey, all study participants consumed starchy staples, but only 16.9%, 3.8%, and 2.7% of them received dark green leafy vegetables, eggs, and organ meat, respectively (**Figure 4**).

Sanitation and hygiene-related characteristics

Three hundred thirty-seven (92.1%) of the study participants got water from protected sources. Less than half, 163(44.5%) of households had a latrine, from which 155(42.3) is functional at the time of the survey. Regarding type of latrine, majority 138(84.7%) is pit type, and the rest 25(15.3) is ventilated improved pit latrine (VIPL) type. Nearly three-fourth, 271(74%) of the study participants dispose of solid waste in open-field outside their compound, while 73(20%) and 22(6%) of the dispose in private pit, and open field in their compound.

All study participants, 366(100%), had the practice of hand-washing before preparing food and before feeding themselves. However, only 71(19.4%) of them used soap for hand-washing (**Figure 5).**

Factors associated with undernutrition

At binary logistic regression material age and education, paternal education and occupation, farming land ownership, maternal ANC follow-up, gravidity, birth interval, getting nutritional counseling, maternal dietary diversity score, and meal frequency were associated with maternal undernutrition at p-value<0.25. But **i**n the final multi-variable logistic regression model, maternal dietary diversity and birth interval were found statistically significantly associated (at the p-value < 0.05) with underweight among lactating mothers. Lactating mothers who didn't meet the MDDS were more than five (AOR= 5.103; 95% CI: 2.128, 12.238) times more likely to be underweight than those who met the MDDS. Mothers with short birth intervals were more than four (AOR=4.800; 95% CI: 2.408, 9.567) time more likely to be undernourished (**Table 5**).

Table 5: Factors associated, at binary and multivariable logistic regression, with undernutrition among lactating mothers in the Abala district, Afar region, northeast Ethiopia				
**Variables / Category**	**Undernutrition (BMI<18.5kg/m^2)**		**COR(95% CI)**	**AOR(95% CI)**
	**Yes**	**No**		
**Maternal age (Years)**				
15-24	28	79	0.54(0.30, 0.97)	0.58(0.18, 1.88)
25-34	53	108	0.74(0.44, 1.25)	0.85(0.37, 1.95)
35-49	39	59	1	1
**Maternal education**				
Not educated	80	143	2.03(0.89, 4.65)	0.59(0.16, 2.15)
Attend primary school	32	74	1.57(0.65, 3.80)	0.56(0.17, 1.90)
Attend secondary & above	8	29	1	1
**Paternal education**				
Not educated	82	146	2.33(1.22, 4.43)	1.96(0.57, 6.76)
Attend primary school	21	39	2.23(1.01, 4.91)	1.69(0.58, 4.92)
Attend secondary & above	14	58	1	1
**Paternal occupation**				
Employed	15	54	1	1
Pastoralist	81	154	1.89(1.01, 3.56)	0.68(0.22, 2.10)
Others	21	35	2.16(0.98, 4.75)	1.68(0.63, 4.47)
**Farming-land ownership**				
Yes	23	83	1	1
No	97	163	2.15(1.27, 3.63)	1.77(0.98, 3.20)
**Maternal ANC follow-up**				
Yes	69	160	1	1
No	51	86	1.38(0.88, 2.15)	0.85(0.48, 1.50)
**Gravidity**				
Primi-gravida	16	51	1	1
2-4	53	116	1.46(0.76, 2.79)	0.48(0.05, 5.15)
$\geq$ 5	51	79	2.06(1.06, 3.99)	0.39(0.03, 4.69)
**Birth interval**				
First birth	16	51	1.80(0.82, 3.94)	1.02(0.10, 10.83)
< 36 month	89	109	4.68(2.53, 8.67)	**4.80(2.41, 9.57)***
$\geq$ 36 months	15	86	1	**1**
**Receiving nutrition counseling**				
Yes	66	162	1	1
No	54	84	1.58(1.01, 2.46)	1.29(0.75, 2.20)
**Maternal minimum dietary diversity score**				
Meet	10	78	1	**1**

Not meet	110	168	5.11(2.53, 10.29)	**5.10(2.13, 12.24)***
**Maternal minimum meal frequency**				
Meet	26	94	1	1
Not meet	94	152	2.24(1.35, 3.70)	1.31(0.70, 2.43)
*significant at P-value<0.001				

Discussion

This study found that the level of undernutrition among lactating mothers is 32.8% (95% CI: 28, 38). This finding was similar to a study conducted in Genta Afeshum woreda of rural Tigray which reported 33.6% [30]. The insignificant difference might be the data collection period for the later study was conducted during Ethiopian Orthodox lent fasting period which might affect dietary feeding practice and nutritional status of the mother, while the study collection period for this study was not on the fasting period.

Compared to the findings of this study, a lower prevalence of undernutrition among lactating mothers was reported in Offa district, Wolyita Zone 15.8% [26], Moyale district, Borena Zone 17.7% [33], Ambo district, West Shewa Zone 21.5% [32], Wombera woreda 25.4% [23], Arba mich Zuria district 26.1% [39], and Raya-Alamata district (Lowland 17.5%, highland 24.6%) [29]. It is also higher than the studies from Nigeria [31] and Zambia [40], in which 4.7% and 3.4% of lactating mothers were undernourished, respectively. The possible reason might be due to the different socio-demographic nature of the area; this study is in the pastoralist community while the other studies were more agrarian.

On the contrary, the finding of this study is lower than the findings from studies done in the Eastern zone of Tigray 38% [34], and Jimma, 40.6% [22]. The possible discrepancy might due to the difference in agro ecologic and cultural variation of the study areas. The other explanation for the observed difference might be the participants included in a study done in Jimma were beneficiaries of the home-based food production and child-centered counseling project.

Maternal dietary diversity score and birth interval were showed a statistically significant association with undernutrition among lactating mothers. Based on this study, lactating mothers who didn’t meet the MDDS were more than five times more likely to be underweight as compared to those who met the MDDS. This is consistent with studies conducted in Jimma [22], Borena [33], and Raya-Alamata districts[29]. This can be explained by dietary diversity is a

proxy indicator of maternal nutrient adequacy, and this nutrient adequacy is essential for protein, lipid, and carbohydrate metabolism [41]. Maternal nutrient needs increase during lactation, and when these needs are not met, they may suffer from malnutrition. During lactation, the energy, protein, and other nutrients in breast milk come from a mother's diet or her body stores [42]. If lactating women do not get enough energy and nutrients they will be at risk of nutrient storage depletion and exacerbates undernutrition [43]. Monotonous diets full of starchy staples, low quantities of fruits and vegetables, and scant animal-source foods result in malnutrition [44, 45].

This study revealed that the probability of being undernutrition is more than four times among lactating mothers who had short birth intervals as compared to mothers who had a long duration of the birth interval. This finding is consistent with studies done in Nigeria [46, 47]. A birth interval of at least 36 months before couples deliver the next child is recommended for mothers and their children's health [48]. Lactation reduces the nutritional reserves of mothers, so longer inter-birth intervals allow for an increase in nutritional reserves [49]. The short birth interval could not give time to the mother to restore her nutritional status, and expose the mother to nutritional stress, morbidity, and mortality [50, 51].

In this study, even though the socio-demographic variables have a significant association at bivariate analysis, they were not significantly associated in the final multivariable model. But some studies [6, 23, 24, 26, 32] showed that family size, maternal education, maternal occupation, farming land ownership, and paternal education and occupation showed a significant association with undernutrition among lactating mothers. Also, contrary to other studies [23] maternal health care characteristics such as teenage pregnancy, ANC follow-up, place of delivery, postnatal care follow-up, maternal meal frequency, nutrition-related education, and maternal workloads had no statistically significant association with maternal undernutrition at multivariable logistic regression model. This difference is might be due to the difference in socio-demographic characteristics of the study areas. This study is conducted in a pastoralist community, while the other studies are mostly agrarian and with better access to health care services.

Strengths of the study

Using primary data, and being a community-based study, which will estimate the true prevalence of the problem.

Limitations of the study

Food consumption patterns might vary seasonally, and the results regarding dietary diversity score and meal frequency are limited to the specific season of the year in which the study was conducted. Those dietary assessments are also recorded using a 24-hour recall method; as a result, they might not reflect the usual intake of an individual. The other limitation of this study is failure to incorporate household food security and wealth index as factors associated with undernutrition which might have also introduced a residual confounding problem;

Conclusions

In this study, nearly one-third of lactating mothers were undernourished, which is higher as compared to other similar studies, and considered as a significant public health problem. Undernutrition was significantly associated with maternal dietary diversity and birth interval. For the prevention of undernutrition among lactating mothers health education on proper and adequate maternal dietary feeding practices, and proper family planning utilization during lactation should be emphasized.

Acknowledgments

We acknowledge the Afar regional and woreda health office heads for their valuable cooperation during the data collection. We would like to extend our gratitude to the data collectors and lactating mothers who participated in this study. Finally, thanks to Samara University for ethical approval and facilitation of this study.

Authors' contribution

Conceptualization: Getahun Fentaw Mulaw

Led data collection and investigation: Getahun Fentaw Mulaw and Kusse Urmale Mare

Formal Analysis and methodology: Getahun Fentaw Mulaw and Kusse Urmale Mare

Writing - original draft: Getahun Fentaw Mulaw

Writing – review and editing: Getahun Fentaw Mulaw and Kusse Urmale Mare, Etsay woldu Anbesu

References

1. Blossner M, De Onis M, Prüss-Üstün A. Malnutrition: quantifying the health impact at national and local levels: World Health Organization; 2005.

2. Unicef. Tracking Progress on Child and Maternal Nutrition: A Survival and Development Priority. eSocialSciences; 2015.

3. Meijers JM, Schols JM, Soeters PB, Halfens RJ. Defining malnutrition: mission or mission impossible? Nutrition. 2010;26(4):432-40.

4. World Health Organization. Progress in partnership: 2017 Progress report on the every woman every child global strategy for Women's, Children's and adolescents' health. World Health Organization; 2017.

5. Black R, Allen L, Bhutta Z, Caulfield L, Onis. Maternal and child undernutrition 1-Maternal and child undernutrition: global and regional exposures and health consequences. Lancet. 2008.

6. Hundera TD, Gemede HF, Wirtu D, Kenie DN. Nutritional status and associated factors among lactating mothers in Nekemte Referral Hospital and Health Centers, Ethiopia. Int J Nutr Food Sci. 2015;4(2):216-22.

7. Walker SP. Nutritional issues for women in developing countries. Proceedings of the Nutrition Society. 1997;56(1B):345-56.

8. Khan Y, Bhutta ZA. Nutritional deficiencies in the developing world: current status and opportunities for intervention. Pediatric Clinics. 2010;57(6):1409-41.

9. Allen LH. B vitamins in breast milk: relative importance of maternal status and intake, and effects on infant status and function. Advances in nutrition. 2012;3(3):362-9.

10. Black RE, Allen LH, Bhutta ZA, Caulfield LE, De Onis M, Ezzati M, et al. Maternal and child undernutrition: global and regional exposures and health consequences. The lancet. 2008;371(9608):243-60.

11. Nakamori M, Ninh NX, Isomura H, Yoshiike N, Hien VTT, Nhug BT, et al. Nutritional status of lactating mothers and their breast milk concentration of iron, zinc and copper in rural Vietnam. Journal of nutritional science and vitaminology. 2009;55(4):338-45.

12. Westspot C. Nutrition in the first 1,000 days: State of the world's mothers 2012. Westport, CT: Save the Children. 2012. ISBN 1-888393-24; 2012.

13. Negash C, Whiting SJ, Henry CJ, Belachew T, Hailemariam TG. Association between maternal and child nutritional status in Hula, rural Southern Ethiopia: A cross sectional study. PloS one. 2015;10(11):e0142301.

14. Ahmed T, Hossain M, Sanin KI. Global burden of maternal and child undernutrition and micronutrient deficiencies. Annals of Nutrition and Metabolism. 2012;61(Suppl. 1):8-17.

15. UNICEF/World Health Organization (WHO)/World Bank Group. Global Nutrition Repot; The burden of malnutrition: Exploring global and regional trends in malnutrition in all its forms; 2020 :

Available from: https://globalnutritionreport.org/reports/global-nutrition-report-2018/burden-malnutrition/

16. Huffman SL, Zehner E, Harvey P, Martin L, Piwoz E, Ndure K, et al. Essential health sector actions to improve maternal nutrition in Africa. LINKAGES Project, Academy for Educational Development. 2001.

17. Achadi E, Ahuja A, Bendech MA, Bhutta ZA, De-Regil LM, Fanzo J, et al. Global nutrition report 2016: From promise to impact: Ending malnutrition by 2030: International Food Policy Research Institute; 2016.

18. Central Statistical Agency (Federal Democratic Republic of Ethiopia). Ethiopian Demographic and Health Survey 2016.

19. Sserwanja Q, Kawuki J. Prevalence of underweight and associated factors among lactating women in Ethiopia: a mini-review. Journal of Advances in Medicine and Medical Research. 2020:1-9.

20. Federal Ministry of Health Adis Ababa (Federal Democratic Republic of Ethiopia). Health Sector Transformation Plan 2015/16–2019/20: Available from: https://www.globalfinancingfacility.org/ethiopia-health-sector-transformation-plan-201516-201920.

21. Wang H, Tesfaye R, NV Ramana G, Chekagn CT. Ethiopia health extension program: an institutionalized community approach for universal health coverage: The World Bank; 2016.

22. Alemayehu M, Argaw A, Mariam AG. Factors associated with malnutrition among lactating women in Subsistence farming households from Dedo and Seqa-Chekorsa districts, Jimma zone, 2014. Developing Country Studies. 2015;5(21):117-8.

23. Berihun S, Kassa GM, Teshome M. Factors associated with underweight among lactating women in Womberma woreda, Northwest Ethiopia; a cross-sectional study. BMC nutrition. 2017;3(1):1-7.

24. Haileslassie K, Mulugeta A, Girma M. Feeding practices, nutritional status and associated factors of lactating women in Samre Woreda, South Eastern Zone of Tigray, Ethiopia. Nutrition journal. 2013;12(1):1-11.

25. Health Department. Abala woreda (Afar region, Ethiopia) helth bureaue report. Juanuary 2020. .

26. Julla BW, Haile A, Ayana G, Eshetu S, Kuche D, Asefa T. Chronic Energy Deficiency and Associated Factors among Lactating Mothers (15-49 years old) in Offa Woreda, Wolayita Zone, SNNPRs, Ethiopia. World Scientific Research. 2018;5(1):13-23.

27. Abeya S, Biru K, Jima A. Factors Associated with Underweight among Lactating Mothers in Adama District, Oromia Region, Ethiopia. J Orthop Bone Res 1: 101 Abstract Keywords: Lactating Women. 2018.

28. Abraham S, Miruts G, Shumye A. Magnitude of chronic energy deficiency and its associated factors among women of reproductive age in the Kunama population, Tigray, Ethiopia, in 2014. BMC nutrition. 2015;1(1):1-9.

29. Sitotaw IK, Hailesslasie K, Adama Y. Comparison of nutritional status and associated factors of lactating women between lowland and highland communities of District Raya, Alamata, Southern Tigiray, Ethiopia. BMC nutrition. 2017;3(1):1-12.

30. Desalegn BB, Lambert C, Riedel S, Negese T, Biesalski HK. Ethiopian orthodox fasting and lactating mothers: Longitudinal study on dietary pattern and nutritional status in rural tigray, Ethiopia. International journal of environmental research and public health. 2018;15(8):1767.

31. Sanusi R, Falana O. The nutritional status of mothers practicing breast feeding in Ibadan, Nigeria. African Journal of Biomedical Research. 2009;12(2):107-12.

32. Zerihun E, Egata G, Mesfin F. Under nutrition and its associated factors among lactating mothers in rural Ambo district, West Shewa zone, Oromia region, Ethiopia. East African Journal of Health and Biomedical Sciences. 2016;1(1):39-48.

33. Bekele H, Jima GH, Regesu AH. Undernutrition and associated factors among lactating women: Community-based cross-sectional study in Moyale District, Borena Zone, Southern Ethiopia. Advances in Public Health. 2020;2020.

34. Abera SF, Kantelhardt EJ, Bezabih AM, Tsadik M, Lauvai J, Ejeta G, et al. What factors are associated with maternal undernutrition in eastern zone of Tigray, Ethiopia? Evidence for nutritional well-being of lactating mothers. BMC public health. 2020;20(1):1-12.

35. Kennedy G, Ballard T, Dop MC. Guidelines for measuring household and individual dietary diversity: Food and Agriculture Organization of the United Nations; 2011.

36. FAO and FHI 360. Minimum dietary diversity for women: a guide for measurement. Rome: FAO. 2016;82.

37. Be CNNDL, Alone MBUDI. 9 Meeting Maternal Nutrient Needs During Lactation.

38. Picciano MF. Pregnancy and lactation: physiological adjustments, nutritional requirements and the role of dietary supplements. The Journal of nutrition. 2003;133(6):1997S-2002S.

39. Tikuye HH, Gebremedhin S, Mesfin A, Whiting S. Prevalence and factors associated with undernutrition among exclusively breastfeeding women in Arba Minch Zuria District, Southern Ethiopia: A cross-sectional community-based study. Ethiopian journal of health sciences. 2019;29(1).

40. Kaliwile C, Michelo C, Titcomb TJ, Moursi M, Donahue Angel M, Reinberg C, et al. Dietary intake patterns among lactating and non-lactating women of reproductive age in rural Zambia. Nutrients. 2019;11(2):288.

41. Arimond M, Wiesmann D, Becquey E, Carriquiry A, Daniels MC, Deitchler M, et al. Simple food group diversity indicators predict micronutrient adequacy of women's diets in 5 diverse, resource-poor settings. The Journal of nutrition. 2010;140(11):2059S-69S.

42. Dewey KG, Cohen RJ, Brown KH, Rivera LL. Effects of exclusive breastfeeding for four versus six months on maternal nutritional status and infant motor development: results of two randomized trials in Honduras. The Journal of nutrition. 2001;131(2):262-7.

43. Dewey KG. Energy and protein requirements during lactation. Annual review of nutrition. 1997;17(1):19-36.

44. Grech AM, Alders R, Darnton-Hill I, Bagnol B, Hikeezi D, O'Leary F. Nutrition Knowledge, Attitudes, and Dietary Intake of Women of Reproductive Age in Bundabunda Ward, Zambia. Clinical Journal of Nutrition and Dietetics. 2018;1(2):1-12.

45. De Bruyn J, Bagnol B, Darnton-Hill I, Maulaga W, Thomson PC, Alders R. Characterising infant and young child feeding practices and the consumption of poultry products in rural Tanzania: A mixed methods approach. Maternal & Child Nutrition. 2018;14(2):e12550.

46. Adebowale S, Adepoju O, Fagbamigbe F. Child spacing and parity progression: Implication for maternal nutritional status among women in Ekiti communities, Southwestern Nigeria. Pakistan Journal of Nutrition. 2011;10(5):485-91.

47. Kathryn G, Roberta J. Birth Spacing Literature: Maternal and Child Nutrition Out Comes. Program in international nutrition, university of Califonia, Davis. 2004.

48. World Health Organization. Report of a WHO technical consultation on birth spacing: Geneva, Switzerland 13-15 June 2005. World Health Organization; 2007.

49. King JC. The risk of maternal nutritional depletion and poor outcomes increases in early or closely spaced pregnancies. The Journal of nutrition. 2003;133(5):1732S-6S.

50. Rutstein S, editor Effect of birth intervals on mortality and health: multivariate cross country analyses. Conference on Optimal Birth Spacing for Central America, Antigua, Guatemala; 2003.

51. Brockman S, Stout I, Marsh K. The Public Health Impact of Optimal Birth Spacing: New Research from Latin America and the Caribbean. 2003.

Point by Point Response to Editorial and reviewers comments (manuscript Id: PONE-D-20-31073)

We appreciate and give thanks for the reviewer(s) and editorials for their positive, constructive comments and suggestions for the improvement of this manuscript. We the authors tried to critically see and incorporate all the given suggestions and comments in the main document (as highlighted by red colors). The grammatical and English language content of the whole manuscript is also revised. The final revised manuscript is uploaded on the online system. We look your interesting response and suggestion.

Comments given by	Specific comments given in each part of the manuscript	Point by point amendments made
Editorial Comments:	• Make the discussion more succinct, some of it is already mentioned in introduction - it should be deleted or should be taken there eg the first para of the discussion.	• The first paragraph of the discussion section is deleted.<br>• The other section of the paragraph is revised.
	• To state the references in the text - use of square brackets	• Changed to square brackets
	• P. 3:" A semi-structured questionnaire was developed through a critical review of relevant literature." Please mention the literature	• A semi-structured questionnaire was developed through a critical review of relevant literature [4, 9, 16, 21, 23-32].
	• Need to check all the Spellers and grammar – e.g p. 5 meat and not meet, multicollinearity etc. English editing is required.	• Edited as Meet, multicollinearity. The whole document is tried to be revised both English editing and grammer.
	• While the nutrition outcome on two important variables - **dietary requirements and birth spacing** are detailed, the discussion should also highlight how socio-demographic variables impact the outcomes	• We tried to discuss the association between dietary diversity and nutritional status of lactating mothers and<br>• The association between birth interval and nutritional status of lactating mothers
	• Could there be a problem of multi-collinearity, or they may be significant at higher p-values.	• Edited as "……But there was no multicollinearity among studied variables at the cut of p-value of 0.05.
	• Please comment on why BMI was preferred to other measures such as MUAC in some other studies. Was the association between MUAC and BMI studied?	• Both the BMI and MUAC were recorded and reported under the result section.<br>• I understand that MUAC is preferred for mothers having

		children less than 6 months of age, and BMI for mothers having > 6months of age children.<br>• But I chose BMI for analysis, because less than 20% of mothers have children less than 6 months of age.<br>• *Rephrased and edited as* “Maternal nutritional status was assessed using both BMI and MUAC. According to BMI, 120(32.8%) of mothers were undernourished; with mild, moderate, and severe degrees of undernourishment occurred in 80(21.9%), 30(8.2%), and 10(2.7%) of respondents, respectively. But using MUAC, 122 (33.3%) of mothers were undernourished. The Pearson’s correlation coefficient(r) between BMI and MUAC was 0.764 (P-value <0.001). The mean (+SD) score of mothers BMI, and MUAC were 20.2(+2.8) kg/m2, 23.9(+2.5) cm, respectively (Table 3 and Figure 2).
	• Abbreviations should be explained whenever used first time in the text, instead of giving in the end.	• We modify. Eg. Adjusted odds Ratio (AOR), Confidence Interval (CI)
	• Generally Ethical statement is a part of methodology	• We incorporated it into methodology section.
	• Funding information should not appear in the Acknowledgments section or other areas of your manuscript. We will only publish funding information present in the Funding Statement section of the online submission form<br>• Please remove any funding-related text from the manuscript and let us know how you would like to update your Funding Statement. Currently, your Funding Statement reads as follows: [The funders had no role in study design, data collection and analysis, decision to publish, or preparation of the manuscript]<br>• Please include your amended statements within your cover letter; we will change the online submission form on your behalf.	• Accepted , Acknowledgments section is revised as follows<br>• **Acknowledgment**: We acknowledge the Afar regional and woreda health office heads for their valuable cooperation during the data collection. We would like to extend our gratitude to the data collectors and lactating mothers participated in this study. Finally, thanks to Samara University for ethical approval and facilitation of this study. “<br>• Funding information is removed from the manuscript, and incorporated in cover later for amendment as follows.<br>• **Funding Statement**: - The funder (Samara University) has no role in designing the study, data collection, data analysis, decision to publish, and preparation of the manuscript.

	• Your ethics statement should only appear in the Methods section of your manuscript. If your ethics statement is written in any section besides the Methods, please move it to the Methods section and delete it from any other section.	• We incorporated it into methodology section, and deleted it from other section
	• Please include your tables as part of your main manuscript and remove the individual files. Please note that supplementary tables (should remain/ be uploaded) as separate "supporting information" files.	• The tables are incorporated within the main manuscript.<br>• The supplementary materials **S1 File** and **S1 Data** are uploaded separately.
	• We suggest you thoroughly copyedit your manuscript for language usage, spelling, and grammar.	• Language, spelling and grammar of the whole manuscript is tried to modified, and amended
	• Please include additional information regarding the survey or questionnaire used in the study and ensure that you have provided sufficient details that others could replicate the analyses. For instance, if you developed a **questionnaire as part of this study** and it is not under a copyright more restrictive than CC-BY, please include a copy, in both the original language and English, as **Supporting Information.** Moreover, please include more details on how the questionnaire was pre-tested, and whether it was validated.	• The questionnaire (both in English and local language) is uploaded as supporting information.<br>• The questionnaire was pretested and validated (this statement is mentioned in the methodology section of the manuscript)
	• In your Methods section, please provide additional information about the **participant recruitment method** and the demographic details of your participants.	• The sampling procedure was a multi-stage sampling technique. Abala district has 14 kebeles from which 5 kebeles were selected randomly. In each selected kebeles, the list of all households having lactating mothers was located (from the health extension worker's family folder). Then the sample-size was proportionally allocated based on the total number of lactating women in each selected kebele. Finally, study participants in each selected kebeles were recruited from the list using a systematic sampling technique, every fixed interval. In

		households where there are more than one lactating women, a lottery method was used to select one participant (Figure 1).
	• Please ensure you have provided sufficient details to replicate the analyses such as:<br>a. The recruitment date range (month and year)	• The study period was from January 5/2020 to February 10/2020.
	b. A description of any inclusion/exclusion criteria that were applied to participant recruitment	• **Eligibility criteria**<br>The source population was all lactating women whose children aged less than 24 months in Abala district. Mothers were included in the study if they were lived in the district for six or more months. Mothers were considered to be excluded if they are seriously ill and physically unable to fit for anthropometric measurements, but no lactating mother was found with such cases. Additionally, women who were or who suspect of pregnancy, according to maternal report, were excluded from the study.
	c. A table of relevant demographic details	• We tried to incorporate some important details in the first paragraph of methodology section.
	d. A statement as to whether your sample can be considered representative of a larger population	• Therefore, the final sample size for this study, to determine both objectives, was taken as 370. This is a minimum representative sample for the study population.
	e. A description of how participants were recruited, and Descriptions of where participants were recruited and where the research took place.	• The sampling procedure was a multi-stage sampling technique. Abala district has 14 kebeles from which 5 kebeles were selected randomly. In each selected kebeles, the list of all households having lactating mothers was located (from the health extension worker's family folder). Then the sample-size was proportionally allocated based on the total number of lactating women in each selected kebele. Finally, study participants in each selected kebeles were recruited from the list using a systematic sampling technique, every fixed interval. In households where there are more than one

			lactating women, a lottery method was used to select one participant (Figure 1).
**Reviewer #1**	**Abstract**	**Background part:**<br>• What means by the sentence ; 'Lactating women from developing countries are nutritionally vulnerable groups because this period places a high nutritional demand on the mother and lead to nutritional stress' i.e is to mean that lactating women from the developed country are not nutritionally vulnerable?	• Modified as follows<br>• Lactating women are nutritionally vulnerable groups because this period places a high nutritional demand on the mother and lead to nutritional stress
		**Methodology part:**<br>• The word methdology is not an appropriate word rather Methods	• Methods
		• Study subjects are not described well i.e. are all lactating mothers or lactating whose children less than 6 months or those who had children 6-24 months age included in your study is not stated vs types of anthropometric measurement used to determine nutritional status	• All lactating mothers having children less than 24 months.<br>• MUAC is best for mothers whose children less than 6 months<br>• BMI is best for mothers whose children aged 6-24 months.<br>• But what we did is we measure both BMI and MUAC for all mothers and we put the results of both. But for association BMI was used, because less than 20% of mothers had children less than 6 months.
		• What means by Study participants were recruited using a multi-stage sampling technique?	• It was to show how we reach the smallest units in which study subjects reside. But we used systematic sampling technique to select study participants.<br>• Rephrased as "Study participants were recruited using a systematic sampling technique."
		• Which types of anthropometric measurements you used is not mentioned? Types of instruments used for anthropometric measurement , sensitivity and specificity of instruments you used should be stated	• Anthropometric measurements (both body mass index and mid upper arm circumference) were taken from each mother using calibrated equipment and standardized techniques.<br>• The weight of lactating mothers was measured using the United Nations International Children's Emergency Fund

			(UNICEF) Electronic Scale with a maximum capacity of 150 kg and an accuracy of 0.1 kg.<br>• Maternal height was measured with standing position using a portable stadiometer to the nearest 0.1 cm<br>• Maternal MUAC was measured using UNICEF measuring tape to the nearest 0.1 cm
		• **General Comments**: Your methods part needs some revision	• Revised according to your recommendation
		**Result part:**<br>• What means by, stunted (height < 145 cm) ? in stating of prevalence of your result part	• We were thinking that to describe stunting of mothers using their height measurement. But based on your recommendation we rephrased it as<br>• “This study showed that 120(32.8%) and 122(33.3%) surveyed mothers were undernourished using a cut off body mass index <18.5 kg/m2 and mid-upper arm circumference <23 Centimeter, respectively.”
	**Background**	• Better if you begin your sentence with definition of your outcome variable	• Modified as follows<br>• Malnutrition is one of the most devastating problems worldwide, and can be manifested as both under nutrition and over nutrition. Under nutrition can be divided in to protein energy malnutrition and micronutrient deficiencies [1]. Under nutrition is the outcome of insufficient food intake, inadequate care, and infectious diseases [2]. Malnutrition among reproductive age women is likely to have a major impact on their own health as well as their children’s health [3, 4].
		• In your fourth paragraph of background section, what mean by the statement ‘significant proportion of lactating mothers are undernourished and maternal mortality ratio is still 412 per 100,000 live births’? how you relate this maternal mortality with under nutrition i.e Is that to mean that this maternal	• It was to mean one of the causes.<br>• Rephrased as “Despite the number of effective and proven nutrition intervention programs in Ethiopia [17, 18], studies [19-21] reported that a significant proportion of lactating mothers are undernourished and it is one of the major cause of maternal morbidity and mortality.”

		mortality is due to under nutrition???	
		• General Comments: Your background section mainly focuses on statements of problem part but it lacks some important points about introduction (background) part	• Based on your recommendation I tried to incorporate the first one paragraph as supportive background section.
	**Methods and materials**	**Study design, setting, and period part:**<br>• Nothing is mentioned about study period. Total population of your study subjects is not indicated	• Accepted and modified<br>• The study period was from January 5/2020 to February 10/2020.<br>• We couldn't get the exact number of lactating mothers in the district, but we put the total estimated population of the district.
		**Eligibility criteria part:**<br>• You stated that respondents who were seriously ill and physically unable to fit for anthropometric measurements. What types of illness and physical deformity you found and excluded? It must be clearly stated.<br>• How you confirm pregnancy to exclude from study?	• Mothers were considered to be excluded if they are seriously ill and physically unable to fit for anthropometric measurements, but no lactating mother was found with such cases. Additionally, women who were or who suspect of pregnancy, according to maternal report, were excluded from the study.<br>• Additionally, women who were or who suspect of pregnancy, **according to maternal report,** were excluded from the study.
		**Sample size determination and sampling procedure:**<br>• Why you used design effect 1.5?<br>• You said that study participants were selected using a systematic sampling technique. If you got the list of lactating mothers from health extension family folder, why you use systematic sampling technique?	• The district has 14 kebeles. From those we selected 5 kebeles. Then from kebeles households with lactating mothers were selected.<br>• Even though it is one district, we assume that there might be some socio-demographic and other variations among kebeles. As a result we used design effect of 1.5.<br>• We used health extension family folder to develop a sampling frame (list of all households in selected kebeles, in which lactating mothers reside). Based on a fixed interval we draw our sampling units. Rephrased as follows

			**Sampling procedure**<br>• The sampling procedure was a multi-stage sampling technique. Abala district has 14 kebeles from which 5 kebeles were selected randomly. In each selected kebeles, the list of all households having lactating mothers was located (from the health extension worker's family folder). Then the sample-size was proportionally allocated based on the total number of lactating women in each selected kebele. Finally, study participants in each selected kebeles were recruited from the list using **a systematic sampling technique**, every fixed interval. In households where there are more than one lactating women, a lottery method was used to select one participant (Figure 1).
		**Data collection procedure and quality control measures part:**<br>• You did not cite any literatures you used for the development of your questionnaire<br>• What is the importance of using 10% of your sample size for pretesting?<br>• Why you used nine food groups for calculation to determine WDDS??? Is it the right way using nine food groups to determine DDS for Women of Reproductive Age???<br>• You said that duplicate anthropometric measurements were done in case of deviations from standard procedures. Which measurements (from this duplicate anthropometric measurements) you documented finally?	• A semi-structured questionnaire was developed through a critical review of relevant literature [5, 18, 21-23, 25, 28-33].<br>• Since it has anthropometric measurement procedures, we wanted our data collectors to be perfect in following the standard procedures.<br>• There are also other options (10 food group category, 12 food group category, 14 food group category). But we considered the 9 food group category will be best for the local context of pastoralist community,(we think that it will be difficult to differentiate some food groups by the community)<br>• We want to say here, if the data collector assumes the first measurement procedure is followed correctly, it was recorded as it is. But during measurement if there is a deviation from the correct procedure, second measurement is considered and recorded. Rephrased as follows"**Standard anthropometric measurements were followed to minimize measurement**

			**errors**.”
		**Data processing and analysis part:**<br>• The final model was tested using Hosmer and Lemeshow chi-square test p-value, and it was P=556, is that P=556???	• Typing error. It was to mean p-value = 0.556
		**Operational definitions part:**<br>• Maternal under-nutrition: Did you use only BMI to determine your outcome variables vs lactating mothers having less than 24 months age children???<br>• Women dietary diversity score (WDDS): Number of food groups used ???<br>• Minimum Meal frequency (MMF) - what is your reference/standard that to be said meal frequency as Meet: > 4 times and Not meet: < 4 times?	• Maternal under-nutrition<br>○ Yes: BMI < 18.5 Kg/m2 and/or MUAC< 23cm<br>○ No: BMI > 18.5 kg/m2 and/or MUAC > 23 cm<br>• There are a lot of options (nine food group category, 10 food group category, 12 food group category)<br>• But we used for this study the nine food group’s category.<br>• There is no standard definition of meal frequency for lactating mothers. But WHO recommends for lactating mothers additional 500 kcal per day. Based on that we decided that a minimum of one densely prepared extra meal will give this calorie.<br>• In Ethiopian context majority of the population consumes 3 times per day. Therefore one additional meal should be added for lactating mothers.
	**Result**	**Socio-demographic characteristics of study participants part:**<br>• Better if you include the response rate<br>• More than half, 223(60.9%) of mothers were not attending formal education- better if you said three-fifth of….	• The response rate was 98.9%..<br>• Three-fifth, 223(60.9%) of mothers were not attending formal education.
		**Maternal nutritional status part:**<br>• Among the 366 respondents, 120(32.8%) of mothers were undernourished; with mild, moderate, and	• Both the BMI and MUAC were recorded and reported under the result section.<br>• I understand that MUAC is preferred for mothers having

		severe degrees of undernourishment occurred in 80(21.9%), 30(8.2%), and 10(2.7%) of respondents, respectively—Did you use only BMI to determine nutritional status i.e did you use BMI for lactating women whose children's age less than 6 months ???	children less than 6 months of age, and BMI for mothers having > 6months of age children.<br>• But I chose BMI for analysis, because less than 20% of mothers have children less than 6 months of age.<br>• ***Rephrased and edited as*** "Maternal nutritional status was assessed using both BMI and MUAC. According to BMI, 120(32.8%) of mothers were undernourished; with mild, moderate, and severe degrees of undernourishment occurred in 80(21.9%), 30(8.2%), and 10(2.7%) of respondents, respectively. But using MUAC, 122 (33.3%) of mothers were undernourished. The Pearson's correlation coefficient(r) between BMI and MUAC was 0.764 (P-value <0.001). The mean (+SD) score of mothers BMI, and MUAC were 20.2(+2.8) kg/m2, 23.9(+2.5) cm, respectively (**Table 3 and Figure 2**).
	**Discussion**	• In the fourth paragraph of your discussion, you said that 'The possible reason for the difference might be the use of different measurement; they use MUAC while this study uses BMI'. What this sentence implies?	• ***Rephrased and discussed as*** "On the contrary, the finding of this study is lower than the findings from studies done in Eastern zone of Tigray 38% [33], and Jimma, 40.6% [21]. The possible discrepancy might due to the difference in socio-demographic characteristics, agro ecologic and cultural variation of the study areas. The other explanation for the observed difference might be the participants included in a study done in Jimma were beneficiaries of the home-based food production and child-cantered counselling project."
		• In the sixth paragraph of your discussion you said, A birth-interval of at least 36 months before couples deliver the next child is recommended for mothers and their children. Is 36 months or 24 months used as recommendation of birth interval in Ethiopian government? Did you use 36 months in your	• World Health Organization (WHO) recommends a minimum **inter-pregnancy interval** of two years or an **inter-birth interval** of thirty-three months or more to ensure the maximum health benefits for the mothers and the new-borns (*World Health Organization: Report of a WHO technical consultation on birth spacing: Geneva, Switzerland 13–15 June 2005. In.: World Health Organization; 2007*).

		analysis?	• In Ethiopian context the minimum inter-pregnancy interval is 24 months, that means the recommended inter-birth interval approaches three years. Therefore, for analysis we used the inter-birth interval of 36 months for this analysis.
		• In the last two paragraphs of your discussion part: please clearly explain the reason how/why this occurred (those variables which is significant in others study but not in your study) and vice versa.	• This difference is might be due to the difference in socio-demographic characteristics of the study areas. This study is conducted in pastoralist community, while the other studies are in mostly agrarian and with better access to health care services.
	**Limitations of the study**	• Being a cross-sectional study- is it limitation? Did not know this (causal relationship) before conducting your study?	• As your recommendation I avoid it. We tried to discuss other limitations
		• Fail to incorporate household food security assessment- Even if you stated as a limitation, this is a major issue that was not be missed especially in accordance with your study subjects and area. Food Security-Major Issue	• Good comment. It was one of our intentions to do so.<br>• We had incorporated it in the study tool. But, there missing value for that particular variable was very high. During supervision we had identified the problem, but the data collectors had told us because of cultural issues and intention of other benefits the mothers were not willing to provide information for that particular variable. That is why we didn't incorporate it in the final analysis.
**Reviewer #2**	The researcher tried to address both the level of under nutrition and some associated factors among pastoralist population who are less advantages as compared the others settings. This is sound good!		
	• However, the sample size determined was only for prevalence and we hesitate that this sample can determine the true predictors of under nutrition. Hence, Authors explanation is required to clear this hesitation.		• ***Accepted, we were removed it to shorten tha manuscript but it was at the proposal and final rapport of the research. Now it is corrected as follows.***<br>• **Sample size for first objective (For assessing prevalence of under nutrition):** The sample size was calculated using a single population proportion formula with a 95%

		confidence level, 5% margin of error, 25% estimated prevalence of under-nutrition (CED) in the study done in Tigray region area (26), and by considering a non-response rate of 10%. **The calculated sample size** for assessing the first objective **was 317.**<br>• **Sample size second objective (to determine factors associated with under nutrition):** The sample size was determined using Epi info version 7 considering the assumptions of 95% Confidence Interval, 80% power and ratio of unexposed to exposed 1:1, assuming different predictors from researches conducted in Ethiopia [23-25]. The predictor variable nutritional advice gives the largest sample size 370 (**Table 1**).
	• The utilization grey literature like office unpublished report should be appropriately addressed based Vancouver system.<br>• The operational definition and measurement issues like Women dietary diversity score (WDDS) described on page 5 should be crystal clear for all readers based on scientific standard.<br>• This is also true for maternal nutritional status measurement described on the same page.	• Accepted, we put the reference in the bibliography.<br>• **Operational definitions**<br>• **Maternal nutritional status**: - is measured using either BMI or MUAC. The mother was considered as<br>○ Undernourished: if her BMI < 18.5 Kg/m^2 and/or MUAC< 23cm<br>○ Not undernourished: if her BMI $\geq$ 18.5 kg/m^2 and/or MUAC $\geq$ 23 cm<br>• **Women dietary diversity score (WDDS):- is calculated**

		**using the** nine food groups' category [34, 35]. The mother was asked the type of food she ate 24-hours preceding the survey regardless of the portion size. WDDS is considered as<br>○ **Low**: if the mother consumed $\leq$ 3 food groups out of the nine food groups<br>○ **Moderate**: if the mother consumed 4-5 food groups out of the nine food groups<br>○ **High**: if the mother consumed $\geq$ 6 food groups out of the nine food groups<br>• **Minimum Meal frequency (MMF)**:- is the frequency or number of meals served by lactating mother 24-hours preceding the survey. The recommended energy intake during the first 6 months of lactation is an additional 500 kilocalorie, which can be achieved by a dense extra meal per day [36, 37].<br>○ **Meet:** $\geq$ 4 times<br>○ **Not meet:** < 4 times
	• Finally, in discussion section some modification needed; particularly for the justifications narrated for discrepancy observed with regards to some findings	• The whole discussion section is tried to modified .
