# Supplementary material for: Nearly one-third of lactating mothers are suffering from undernutrition in pastoral community, Afar region, Ethiopia: Community-based cross-sectional study
Source: PLoS One. 2021 Jul 9;16(7):e0254075. doi: 10.1371/journal.pone.0254075 (PMC8270417; doi:10.1371/journal.pone.0254075)
Supplement: Attachment — Submitted filename: Response to Reviewers.docx [file pone.0254075.s003.docx]

**Point by Point Response to Editorial and reviewers comments (manuscript Id: PONE-D-20-31073)**

We appreciate and give thanks for the reviewer(s) and editorials for their positive, constructive comments and suggestions for the improvement of this manuscript. We the authors tried to critically see and incorporate all the given suggestions and comments in the main document (as highlighted by red colors). The grammatical and English language content of the whole manuscript is also revised. The final revised manuscript is uploaded on the online system. We look your interesting response and suggestion.

| Comments given by | Specific comments given in each part of the manuscript | | Point by point amendments made |
| --- | --- | --- | --- |
| Editorial Comments: | - Make the discussion more succinct, some of it is already mentioned in introduction - it should be deleted or should be taken there eg the first para of the discussion. | | - The first paragraph of the discussion section is deleted. - The other section of the paragraph is revised. |
|  | - To state the references in the text - use of square brackets | | - Changed to square brackets |
|  | - P. 3:” A semi-structured questionnaire was developed through a critical review of relevant literature.” Please mention the literature | | - A semi-structured questionnaire was developed through a critical review of relevant literature [4, 9, 16, 21, 23-32]. |
|  | - Need to check all the Spellers and grammar – e.g p. 5 meat and not meet, multicollinearity etc. English editing is required. | | - Edited as Meet, multicollinearity. The whole document is tried to be revised both English editing and grammer. |
|  | - While the nutrition outcome on two important variables - **dietary requirements and birth spacing** are detailed, the discussion should also highlight how socio-demographic variables impact the outcomes | | - We tried to discuss the association between dietary diversity and nutritional status of lactating mothers and - The association between birth interval and nutritional status of lactating mothers |
|  | - Could there be a problem of multi-collinearity, or they may be significant at higher p-values. | | - Edited as “……But there was no multicollinearity among studied variables at the cut of p-value of 0.05. |
|  | - Please comment on why BMI was preferred to other measures such as MUAC in some other studies. Was the association between MUAC and BMI studied? | | - Both the BMI and MUAC were recorded and reported under the result section. - I understand that MUAC is preferred for mothers having children less than 6 months of age, and BMI for mothers having > 6months of age children. - But I chose BMI for analysis, because less than 20% of mothers have children less than 6 months of age. - *Rephrased and edited as* “Maternal nutritional status was assessed using both BMI and MUAC. According to BMI, 120(32.8%) of mothers were undernourished; with mild, moderate, and severe degrees of undernourishment occurred in 80(21.9%), 30(8.2%), and 10(2.7%) of respondents, respectively. But using MUAC, 122 (33.3%) of mothers were undernourished. The Pearson’s correlation coefficient(r) between BMI and MUAC was 0.764 (P-value <0.001). The mean (+SD) score of mothers BMI, and MUAC were 20.2(+2.8) kg/m2, 23.9(+2.5) cm, respectively (Table 3 and Figure 2). |
|  | - Abbreviations should be explained whenever used first time in the text, instead of giving in the end. | | - We modify. Eg. Adjusted odds Ratio (AOR), Confidence Interval (CI) |
|  | - Generally Ethical statement is a part of methodology | | - We incorporated it into methodology section. |
|  | - Funding information should not appear in the Acknowledgments section or other areas of your manuscript. We will only publish funding information present in the Funding Statement section of the online submission form - Please remove any funding-related text from the manuscript and let us know how you would like to update your Funding Statement. Currently, your Funding Statement reads as follows: [The funders had no role in study design, data collection and analysis, decision to publish, or preparation of the manuscript] - Please include your amended statements within your cover letter; we will change the online submission form on your behalf. | | - Accepted , Acknowledgments section is revised as follows - **Acknowledgment**: We acknowledge the Afar regional and woreda health office heads for their valuable cooperation during the data collection. We would like to extend our gratitude to the data collectors and lactating mothers participated in this study. Finally, thanks to Samara University for ethical approval and facilitation of this study. “ - Funding information is removed from the manuscript, and incorporated in cover later for amendment as follows. - **Funding Statement**: - The funder (Samara University) has no role in designing the study, data collection, data analysis, decision to publish, and preparation of the manuscript. |
|  | - Your ethics statement should only appear in the Methods section of your manuscript. If your ethics statement is written in any section besides the Methods, please move it to the Methods section and delete it from any other section. | | - We incorporated it into methodology section, and deleted it from other section |
|  | - Please include your tables as part of your main manuscript and remove the individual files. Please note that supplementary tables (should remain/ be uploaded) as separate "supporting information" files. | | - The tables are incorporated within the main manuscript. - The supplementary materials **S1 File** and **S1 Data** are uploaded separately. |
|  | - We suggest you thoroughly copyedit your manuscript for language usage, spelling, and grammar. | | - Language, spelling and grammar of the whole manuscript is tried to modified, and amended |
|  | - Please include additional information regarding the survey or questionnaire used in the study and ensure that you have provided sufficient details that others could replicate the analyses. For instance, if you developed a **questionnaire as part of this study** and it is not under a copyright more restrictive than CC-BY, please include a copy, in both the original language and English, as **Supporting Information.** Moreover, please include more details on how the questionnaire was pre-tested, and whether it was validated. | | - The questionnaire (both in English and local language) is uploaded as supporting information. - The questionnaire was pretested and validated (this statement is mentioned in the methodology section of the manuscript) |
|  | - In your Methods section, please provide additional information about the **participant recruitment method** and the demographic details of your participants. | | - The sampling procedure was a multi-stage sampling technique. Abala district has 14 kebeles from which 5 kebeles were selected randomly. In each selected kebeles, the list of all households having lactating mothers was located (from the health extension worker’s family folder). Then the sample-size was proportionally allocated based on the total number of lactating women in each selected kebele. Finally, study participants in each selected kebeles were recruited from the list using a systematic sampling technique, every fixed interval. In households where there are more than one lactating women, a lottery method was used to select one participant (Figure 1). |
|  | - Please ensure you have provided sufficient details to replicate the analyses such as:   a. The recruitment date range (month and year) | | - The study period was from January 5/2020 to February 10/2020. |
|  | b. A description of any inclusion/exclusion criteria that were applied to participant recruitment | | - **Eligibility criteria**   The source population was all lactating women whose children aged less than 24 months in Abala district. Mothers were included in the study if they were lived in the district for six or more months. Mothers were considered to be excluded if they are seriously ill and physically unable to fit for anthropometric measurements, but no lactating mother was found with such cases. Additionally, women who were or who suspect of pregnancy, according to maternal report, were excluded from the study. |
|  | c. A table of relevant demographic details | | - We tried to incorporate some important details in the first paragraph of methodology section. |
|  | d. A statement as to whether your sample can be considered representative of a larger population | | - Therefore, the final sample size for this study, to determine both objectives, was taken as 370. This is a minimum representative sample for the study population. |
|  | e. A description of how participants were recruited, and Descriptions of where participants were recruited and where the research took place. | | - The sampling procedure was a multi-stage sampling technique. Abala district has 14 kebeles from which 5 kebeles were selected randomly. In each selected kebeles, the list of all households having lactating mothers was located (from the health extension worker’s family folder). Then the sample-size was proportionally allocated based on the total number of lactating women in each selected kebele. Finally, study participants in each selected kebeles were recruited from the list using a systematic sampling technique, every fixed interval. In households where there are more than one lactating women, a lottery method was used to select one participant (Figure 1). |
| **Reviewer #1** | **Abstract** | **Background part:**   - What means by the sentence ; ‘Lactating women from developing countries are nutritionally vulnerable groups because this period places a high nutritional demand on the mother and lead to nutritional stress’ i.e is to mean that lactating women from the developed country are not nutritionally vulnerable? | - Modified as follows - Lactating women are nutritionally vulnerable groups because this period places a high nutritional demand on the mother and lead to nutritional stress |
|  |  | **Methodology part:**   - The word methdology is not an appropriate word rather Methods | - Methods |
|  |  | - Study subjects are not described well i.e. are all lactating mothers or lactating whose children less than 6 months or those who had children 6-24 months age included in your study is not stated vs types of anthropometric measurement used to determine nutritional status | - All lactating mothers having children less than 24 months. - MUAC is best for mothers whose children less than 6 months - BMI is best for mothers whose children aged 6-24 months. - But what we did is we measure both BMI and MUAC for all mothers and we put the results of both. But for association BMI was used, because less than 20% of mothers had children less than 6 months. |
|  |  | - What means by Study participants were recruited using a multi-stage sampling technique? | - It was to show how we reach the smallest units in which study subjects reside. But we used systematic sampling technique to select study participants. - Rephrased as “Study participants were recruited using a systematic sampling technique.” |
|  |  | - Which types of anthropometric measurements you used is not mentioned? Types of instruments used for anthropometric measurement , sensitivity and specificity of instruments you used should be stated | - Anthropometric measurements (both body mass index and mid upper arm circumference) were taken from each mother using calibrated equipment and standardized techniques. - The weight of lactating mothers was measured using the United Nations International Children's Emergency Fund (UNICEF) Electronic Scale with a maximum capacity of 150 kg and an accuracy of 0.1 kg. - Maternal height was measured with standing position using a portable stadiometer to the nearest 0.1 cm - Maternal MUAC was measured using UNICEF measuring tape to the nearest 0.1 cm |
|  |  | - **General Comments**: Your methods part needs some revision | - Revised according to your recommendation |
|  |  | **Result part:**   - What means by, stunted (height < 145 cm) ? in stating of prevalence of your result part | - We were thinking that to describe stunting of mothers using their height measurement. But based on your recommendation we rephrased it as - “This study showed that 120(32.8%) and 122(33.3%) surveyed mothers were undernourished using a cut off body mass index <18.5 kg/m2 and mid-upper arm circumference <23 Centimeter, respectively.” |
|  | **Background** | - Better if you begin your sentence with definition of your outcome variable | - Modified as follows - Malnutrition is one of the most devastating problems worldwide, and can be manifested as both under nutrition and over nutrition. Under nutrition can be divided in to protein energy malnutrition and micronutrient deficiencies [1]. Under nutrition is the outcome of insufficient food intake, inadequate care, and infectious diseases [2]. Malnutrition among reproductive age women is likely to have a major impact on their own health as well as their children’s health [3, 4]. |
|  |  | - In your fourth paragraph of background section, what mean by the statement ‘significant proportion of lactating mothers are undernourished and maternal mortality ratio is still 412 per 100,000 live births’? how you relate this maternal mortality with under nutrition i.e Is that to mean that this maternal mortality is due to under nutrition??? | - It was to mean one of the causes. - Rephrased as “Despite the number of effective and proven nutrition intervention programs in Ethiopia [17, 18], studies [19-21] reported that a significant proportion of lactating mothers are undernourished and it is one of the major cause of maternal morbidity and mortality.” |
|  |  | - General Comments: Your background section mainly focuses on statements of problem part but it lacks some important points about introduction (background) part | - Based on your recommendation I tried to incorporate the first one paragraph as supportive background section. |
|  | **Methods and materials** | **Study design, setting, and period part:**   - Nothing is mentioned about study period. Total population of your study subjects is not indicated | - Accepted and modified - The study period was from January 5/2020 to February 10/2020. - We couldn’t get the exact number of lactating mothers in the district, but we put the total estimated population of the district. |
|  |  | **Eligibility criteria part:**   - You stated that respondents who were seriously ill and physically unable to fit for anthropometric measurements. What types of illness and physical deformity you found and excluded? It must be clearly stated. - How you confirm pregnancy to exclude from study? | - Mothers were considered to be excluded if they are seriously ill and physically unable to fit for anthropometric measurements, but no lactating mother was found with such cases. Additionally, women who were or who suspect of pregnancy, according to maternal report, were excluded from the study. - Additionally, women who were or who suspect of pregnancy, **according to maternal report,** were excluded from the study. |
|  |  | **Sample size determination and sampling procedure:**   - Why you used design effect 1.5? - You said that study participants were selected using a systematic sampling technique. If you got the list of lactating mothers from health extension family folder, why you use systematic sampling technique? | - The district has 14 kebeles. From those we selected 5 kebeles. Then from kebeles households with lactating mothers were selected. - Even though it is one district, we assume that there might be some socio-demographic and other variations among kebeles. As a result we used design effect of 1.5. - We used health extension family folder to develop a sampling frame (list of all households in selected kebeles, in which lactating mothers reside). Based on a fixed interval we draw our sampling units. Rephrased as follows   **Sampling procedure**   - The sampling procedure was a multi-stage sampling technique. Abala district has 14 kebeles from which 5 kebeles were selected randomly. In each selected kebeles, the list of all households having lactating mothers was located (from the health extension worker’s family folder). Then the sample-size was proportionally allocated based on the total number of lactating women in each selected kebele. Finally, study participants in each selected kebeles were recruited from the list using **a systematic sampling technique**, every fixed interval. In households where there are more than one lactating women, a lottery method was used to select one participant (Figure 1). |
|  |  | **Data collection procedure and quality control measures part:**   - You did not cite any literatures you used for the development of your questionnaire - What is the importance of using 10% of your sample size for pretesting? - Why you used nine food groups for calculation to determine WDDS??? Is it the right way using nine food groups to determine DDS for Women of Reproductive Age??? - You said that duplicate anthropometric measurements were done in case of deviations from standard procedures. Which measurements (from this duplicate anthropometric measurements) you documented finally? | - A semi-structured questionnaire was developed through a critical review of relevant literature [5, 18, 21-23, 25, 28-33]. - Since it has anthropometric measurement procedures, we wanted our data collectors to be perfect in following the standard procedures. - There are also other options (10 food group category, 12 food group category, 14 food group category). But we considered the 9 food group category will be best for the local context of pastoralist community,(we think that it will be difficult to differentiate some food groups by the community) - We want to say here, if the data collector assumes the first measurement procedure is followed correctly, it was recorded as it is. But during measurement if there is a deviation from the correct procedure, second measurement is considered and recorded. Rephrased as follows”**Standard anthropometric measurements were followed to minimize measurement errors**.” |
|  |  | **Data processing and analysis part:**   - The final model was tested using Hosmer and Lemeshow chi-square test p-value, and it was P=556, is that P=556??? | - Typing error. It was to mean p-value = 0.556 |
|  |  | **Operational definitions part:**   - Maternal under-nutrition: Did you use only BMI to determine your outcome variables vs lactating mothers having less than 24 months age children??? - Women dietary diversity score (WDDS): Number of food groups used ??? - Minimum Meal frequency (MMF) - what is your reference/standard that to be said meal frequency as Meet: > 4 times and Not meet: < 4 times? | - Maternal under-nutrition   - Yes: BMI < 18.5 Kg/m2 and/or MUAC< 23cm   - No: BMI > 18.5 kg/m2 and/or MUAC > 23 cm - There are a lot of options (nine food group category, 10 food group category, 12 food group category) - But we used for this study the nine food group’s category. - There is no standard definition of meal frequency for lactating mothers. But WHO recommends for lactating mothers additional 500 kcal per day. Based on that we decided that a minimum of one densely prepared extra meal will give this calorie. - In Ethiopian context majority of the population consumes 3 times per day. Therefore one additional meal should be added for lactating mothers. |
|  | **Result** | **Socio-demographic characteristics of study participants part:**   - Better if you include the response rate - More than half, 223(60.9%) of mothers were not attending formal education- better if you said three-fifth of…. | - The response rate was 98.9%.. - Three-fifth, 223(60.9%) of mothers were not attending formal education. |
|  |  | **Maternal nutritional status part:**   - Among the 366 respondents, 120(32.8%) of mothers were undernourished; with mild, moderate, and severe degrees of undernourishment occurred in 80(21.9%), 30(8.2%), and 10(2.7%) of respondents, respectively—Did you use only BMI to determine nutritional status i.e did you use BMI for lactating women whose children’s age less than 6 months ??? | - Both the BMI and MUAC were recorded and reported under the result section. - I understand that MUAC is preferred for mothers having children less than 6 months of age, and BMI for mothers having > 6months of age children. - But I chose BMI for analysis, because less than 20% of mothers have children less than 6 months of age. - ***Rephrased and edited as*** “Maternal nutritional status was assessed using both BMI and MUAC. According to BMI, 120(32.8%) of mothers were undernourished; with mild, moderate, and severe degrees of undernourishment occurred in 80(21.9%), 30(8.2%), and 10(2.7%) of respondents, respectively. But using MUAC, 122 (33.3%) of mothers were undernourished. The Pearson’s correlation coefficient(r) between BMI and MUAC was 0.764 (P-value <0.001). The mean (+SD) score of mothers BMI, and MUAC were 20.2(+2.8) kg/m2, 23.9(+2.5) cm, respectively (**Table 3 and Figure 2**). |
|  | **Discussion** | - In the fourth paragraph of your discussion, you said that ‘The possible reason for the difference might be the use of different measurement; they use MUAC while this study uses BMI’. What this sentence implies? | - ***Rephrased and discussed as*** “On the contrary, the finding of this study is lower than the findings from studies done in Eastern zone of Tigray 38% [33], and Jimma, 40.6% [21]. The possible discrepancy might due to the difference in socio-demographic characteristics, agro ecologic and cultural variation of the study areas. The other explanation for the observed difference might be the participants included in a study done in Jimma were beneficiaries of the home-based food production and child-cantered counselling project.” |
|  |  | - In the sixth paragraph of your discussion you said, A birth-interval of at least 36 months before couples deliver the next child is recommended for mothers and their children. Is 36 months or 24 months used as recommendation of birth interval in Ethiopian government? Did you use 36 months in your analysis? | - World Health Organization (WHO) recommends a minimum **inter-pregnancy interval** of two years or an **inter-birth interval** of thirty-three months or more to ensure the maximum health benefits for the mothers and the new-borns (*World Health Organization: Report of a WHO technical consultation on birth spacing: Geneva, Switzerland 13–15 June 2005. In.: World Health Organization; 2007*). - In Ethiopian context the minimum inter-pregnancy interval is 24 months, that means the recommended inter-birth interval approaches three years. Therefore, for analysis we used the inter-birth interval of 36 months for this analysis. |
|  |  | - In the last two paragraphs of your discussion part: please clearly explain the reason how/why this occurred (those variables which is significant in others study but not in your study) and vice versa. | - This difference is might be due to the difference in socio-demographic characteristics of the study areas. This study is conducted in pastoralist community, while the other studies are in mostly agrarian and with better access to health care services. |
|  | **Limitations of the study** | - Being a cross-sectional study- is it limitation? Did not know this (causal relationship) before conducting your study? | - As your recommendation I avoid it. We tried to discuss other limitations |
|  |  | - Fail to incorporate household food security assessment- Even if you stated as a limitation, this is a major issue that was not be missed especially in accordance with your study subjects and area. Food Security-Major Issue | - Good comment. It was one of our intentions to do so. - We had incorporated it in the study tool. But, there missing value for that particular variable was very high. During supervision we had identified the problem, but the data collectors had told us because of cultural issues and intention of other benefits the mothers were not willing to provide information for that particular variable. That is why we didn’t incorporate it in the final analysis. |
| **Reviewer #2** | The researcher tried to address both the level of under nutrition and some associated factors among pastoralist population who are less advantages as compared the others settings. This is sound good! | | |
|  | - However, the sample size determined was only for prevalence and we hesitate that this sample can determine the true predictors of under nutrition. Hence, Authors explanation is required to clear this hesitation. | | - ***Accepted, we were removed it to shorten tha manuscript but it was at the proposal and final rapport of the research. Now it is corrected as follows.*** - **Sample size for first objective (For assessing prevalence of under nutrition):** The sample size was calculated using a single population proportion formula with a 95% confidence level, 5% margin of error, 25% estimated prevalence of under-nutrition (CED) in the study done in Tigray region area (26), and by considering a non-response rate of 10%. **The calculated sample size** for assessing the first objective **was 317.** - **Sample size second objective (to determine factors associated with under nutrition):** The sample size was determined using Epi info version 7 considering the assumptions of 95% Confidence Interval, 80% power and ratio of unexposed to exposed 1:1, assuming different predictors from researches conducted in Ethiopia [23-25]. The predictor variable nutritional advice gives the largest sample size 370 (**Table 1**). |
|  | - The utilization grey literature like office unpublished report should be appropriately addressed based Vancouver system. - The operational definition and measurement issues like Women dietary diversity score (WDDS) described on page 5 should be crystal clear for all readers based on scientific standard. - This is also true for maternal nutritional status measurement described on the same page. | | - Accepted, we put the reference in the bibliography. - **Operational definitions** - **Maternal nutritional status**: - is measured using either BMI or MUAC. The mother was considered as   - Undernourished: if her BMI < 18.5 Kg/m^2^ and/or MUAC< 23cm   - Not undernourished: if her BMI ≥ 18.5 kg/m^2^ and/or MUAC ≥ 23 cm - **Women dietary diversity score (WDDS):- is calculated using the** nine food groups’ category [34, 35]. The mother was asked the type of food she ate 24-hours preceding the survey regardless of the portion size. WDDS is considered as - **Low**: if the mother consumed ≤ 3 food groups out of the nine food groups - **Moderate**: if the mother consumed 4-5 food groups out of the nine food groups - **High**: if the mother consumed ≥ 6 food groups out of the nine food groups - **Minimum Meal frequency (MMF)**:- is the frequency or number of meals served by lactating mother 24-hours preceding the survey. The recommended energy intake during the first 6 months of lactation is an additional 500 kilocalorie, which can be achieved by a dense extra meal per day [36, 37].   - **Meet:** ≥ 4 times   - **Not meet:** < 4 times |
|  | - Finally, in discussion section some modification needed; particularly for the justifications narrated for discrepancy observed with regards to some findings | | - The whole discussion section is tried to modified . |
